# Long-term impact of giving antibiotics before skin incision versus after cord clamping on children born by caesarean section: protocol for a longitudinal study based on UK electronic health records

**DOI:** 10.1136/bmjopen-2019-033013

**Published:** 2019-09-26

**Authors:** Dana Šumilo, Krishnarajah Nirantharakumar, Brian H Willis, Gavin Rudge, James Martin, Krishna Gokhale, Rasiah Thayakaran, Nicola J Adderley, Joht Singh Chandan, Kelvin Okoth, Ruth Hewston, Magdalena Skrybant, Jonathan J Deeks, Peter Brocklehurst

**Affiliations:** 1 Institute of Applied Health Research, University of Birmingham, Birmingham, UK; 2 Midlands Health Data Research UK, University of Birmingham, Birmingham, UK; 3 Patient and Public Contributor, Birmingham, UK; 4 NIHR Birmingham Biomedical Research Centre, University Hospitals Birmingham NHS Foundation Trust and University of Birmingham, Birmingham, UK

**Keywords:** caesarean section, antibiotic prophylaxis, child, asthma, eczema, immune system diseases

## Abstract

**Introduction:**

In the UK, about a quarter of women give birth by caesarean section (CS) and are offered prophylactic broad-spectrum antibiotics to reduce the risk of maternal postpartum infection. In 2011, national guidance was changed from recommending antibiotics after the umbilical cord was cut to giving antibiotics prior to skin incision based on evidence that earlier administration reduces maternal infectious morbidity. Although antibiotics cross the placenta, there are no known short-term harms to the baby. This study aims to address the research gap on longer term impact of these antibiotics on child health.

**Methods and analysis:**

A controlled interrupted time series study will use anonymised mother-baby linked routine electronic health records for children born during 2006–2018 recorded in UK primary care (The Health Improvement Network, THIN and Clinical Practice Research Datalink, CPRD) and secondary care (Hospital Episode Statistics, HES) databases. The primary outcomes of interest are asthma and eczema, two common allergy-related diseases in childhood. In-utero exposure to antibiotics immediately prior to CS will be compared with no exposure when given after cord clamping. The risk of outcomes in children delivered by CS will also be compared with a control cohort delivered vaginally to account for time effects. We will use all available data from THIN, CPRD and HES with estimated power of 80% and 90% to detect relative increase in risk of asthma of 16% and 18%, respectively at the 5% significance level.

**Ethics and dissemination:**

Ethical approval has been obtained from the University of Birmingham Ethical Review Committee with scientific approvals obtained from the independent scientific advisory committees from the Medicines and Healthcare products Regulatory Agency for CPRD and the data provider, IQVIA for THIN. The results will be published in peer-reviewed journals, presented at national and international conferences and disseminated to stakeholders.

**Strengths and limitations of this study**

- A large sample size including mother-child linked data from two nationally representative primary healthcare databases and a secondary healthcare database.
- Investigation of a broad range of relevant child outcomes including severity.
- Investigation of maternal outcomes using real world evidence to confirm findings reported in randomised controlled trials.
- Use of a comparison group of vaginally delivered children to effectively control for changes in diagnosis, recording and exposures over time.
- Timing of prophylactic antibiotic administration is not recorded in routine healthcare data; therefore, analysis is based on the estimated proportion of hospitals with the preincision antibiotic policy in each year during the study period.

## Introduction

Births by caesarean section (CS) account for over 20% of births globally and are increasing.^1^ Over one in four babies in the UK are born by CS.^2–5^ CS is a surgical procedure and women undergoing CS are at increased risk of developing infections after giving birth which can be prevented by prophylactic antibiotics. Before 2011, the national guidance advised administering intravenous prophylactic antibiotics for women undergoing CS after the baby’s cord had been clamped to prevent exposing the baby to antibiotics. In 2011, the guidance was changed to recommend giving antibiotics to women undergoing CS prior to skin incision. This was based on evidence that earlier administration reduces maternal infectious morbidity.^6^ The current Cochrane review summarises data from 10 randomised trials (5041 women) which showed a near halving of risk of all postpartum maternal infection (43%, 95% CI 28% to 55%), endometritis (46%, CI 21% to 64%) and wound infection (41%, CI 19% to 56%) compared with giving antibiotics after clamping the baby’s umbilical cord.^7^ Most postpartum maternal infections, however, are mild and respond well to treatment.^8^

Preoperative prophylactic antibiotics rapidly cross the placenta exposing babies to high dose broad spectrum antibiotics around the time of birth.^9^ Although no short-term harms to the baby have been reported,^6^ intrapartum antibiotics have been shown to alter the gut microbiota of newborns.^10^ There is growing evidence that the composition of gut microbes in infants plays a role in their immune system development including response to different antigens and inflammation and is associated with susceptibility to asthma, allergies and other immune-related diseases later in life.^11–18^ There is a paucity of research regarding the longer term effect of preincision prophylactic antibiotics for CS on child health.

### Aim

The overall aim of this research study is to investigate whether the change in the guidance from recommending prophylactic antibiotics after cord clamping to preincision antibiotics has had any effect on the incidence of allergic and other related health conditions in children born by CS in the UK. This study will provide evidence on long-term impacts of CS preoperative prophylactic antibiotics to inform current guidance regarding the timing of administration of these antibiotics. It will either reinforce the current recommendation or, if negative impacts on child health are observed, will enable assessment of the magnitude of the risks against the benefits of reduced maternal morbidity.

### Objectives

The primary objective of the study is to investigate whether preincisional in-utero exposure to antibiotics immediately prior to birth (Intervention) compared with no preincisional antibiotic exposure (Comparator) increases the risk of 1) asthma and 2) eczema (Outcomes) in children born by CS (Population). The relationship between antibiotic exposure and asthma and eczema severity (defined based on prescribing information and hospital admission data) will also be explored.

Secondary objectives:

1. Investigating the effect of preincision prophylactic antibiotics in children born by CS on: a) other allergic and allergy-related diseases; b) autoimmune diseases; c) infections and inflammation; d) other immune system-related conditions; e) neurodevelopmental conditions; f) less specific measures of child health (colic and failure to thrive).
2. Investigating the effect of preincision prophylactic antibiotics in children born by CS on health service utilisation (overall consultation frequency in primary care and hospital admissions).
3. Investigating if the effects of a reduction in postpartum maternal infectious morbidity shown in randomised controlled trials outside the UK can be replicated in the UK using routine healthcare data.

## Methods and analysis

### Study design

To address the primary objective and secondary objectives 1 and 2, a controlled interrupted time series study will be undertaken using a cohort of women and their children born between 2006 and 2018 in the UK who are included in two routine primary care databases, The Health Improvement Network (THIN) or Clinical Practice Research Datalink (CPRD), and the secondary care HES database.

### Target population

Children born by CS and exposed, in utero, to antibiotics immediately prior to birth will be compared with children born by CS and not exposed, in utero, to antibiotics immediately prior to birth. Children born vaginally during the same time period will be included as a control group.

### Eligibility criteria

All liveborn children for whom the birth year is between 2006 and 2018 will be included; the child and their mother’s healthcare record can be linked in primary care (THIN or CPRD) or secondary care (HES) databases; the mode of delivery, CS or vaginal delivery (VD), can be identified based on recording in primary care (THIN, CPRD) and/or secondary care (HES).

### Exclusion criteria

Children with missing delivery information will be excluded. In case of multiple births (eg, twins), one of the children will be randomly selected for inclusion to ensure independence of observations.

### Study outcomes

The primary outcomes for the study are the incidence of (1) asthma and (2) eczema. The main analysis for primary outcomes will be done separately in the primary care dataset and the secondary care (HES) dataset (the latter including only hospitals for which the year of antibiotic prescribing policy change is known).

Secondary outcomes are other allergic and allergy-related diseases, autoimmune diseases, infections and inflammation, other immune system-related conditions, neurodevelopmental conditions, less specific measures of child health, healthcare utilisation and maternal postpartum infectious morbidity (table 1).

**Table 1:** The list of secondary outcomes

Outcome	Corresponding secondary objective	Datasets analysed	
		Primary care	Secondary care
*Health conditions and symptoms in children*	1.		
Other allergic and allergy-related conditions:	1.a		
Food allergy/intolerance		x	
Allergic rhinitis and conjunctivitis		x	
>1 allergy related disease (asthma, eczema, food allergy/intolerance, allergic rhinitis and conjunctivitis)		x	
Penicillin allergy*		x	
Anaphylaxis*		x	x
High risk of anaphylactic reaction (prescribing of automatic injection devices containing epinephrine)*		x	
Autoimmune diseases:	1.b		
Type 1 diabetes*		x	x
Coeliac disease*		x	x
Juvenile idiopathic arthritis*		x	x
Scleroderma/systemic sclerosis*†		x	x
Inflammatory myopathies*†		x	x
SLE*†		x	x
Autoimmune (idiopathic) ITP*		x	x
Juvenile pernicious (megaloblastic) anaemia*		x	x
Childhood vitiligo*†		x	
Infections and inflammation:	1 .c		
Neonatal sepsis (early and late onset)			x
Other sepsis*			x
Wheeze		x	
Upper respiratory tract infections*		x	
Lower respiratory tract infections*		x	x
Bronchiolitis*		x	x
Gastroenteritis*		x	x
Inflammatory bowel disease†		x	x
Urinary tract infections*		x	x
Antibiotic prescribing*		x	
Other immune system-related conditions:	1.d		
Necrotising enterocolitis			x
Leukaemia*†		x	x
Neurodevelopmental conditions:	1.e		
Cerebral palsy		x	
Autism spectrum disorder*		x	
ADHD*		x	
Less specific measures of child health:	1 .f		
Colic*		x	
Failure to thrive*		x	
*Healthcare utilisation in children*	2.		
Primary care consultations*		x	
Hospital admissions*			x
*Maternal outcomes (6 weeks post partum)*	3.		
Composite infectious morbidity (wound infection, endometritis/endomyometritis, pelvic abscess, maternal sepsis, death attributed to infection)		x	x
Endometritis/endomyometritis		x	x
Wound infection		x	x
Urinary tract infection/cystitis/pyelonephritis		x	x
Sepsis		x	x
Pelvic abscess		x	x
Maternal death (if infection related)*†			x
Antibiotic prescribing*		x	
Length of hospital stay*			x

*Exploratory outcome due to insufficient evidence base, including lack of longitudinal studies investigating the association between microbiota/early antibiotic exposure and outcome of interest;

†Tabulation if the outcome is very rare.

ADHD, attention deficit hyperactivity disorder; ITP, thrombocytopenic purpura; SLE, systemic lupus erythematosus.

### Data sources

To maximise the sample size, we will combine two UK-wide primary care research databases, THIN and CPRD, containing anonymised patient records of over 10% of the UK patient population.^19^ Both databases are broadly generalisable to the UK population in terms of demographics and medical condition prevalence.^20 21^ There is overlap between the databases at general practice level, with THIN and CPRD containing 37% and 46% unique practices, respectively. The databases do not use the same identifiers for patients or practices, but the overlapping practices can be identified reliably using patient registration, demographic and medical record information and the duplicates removed to create a combined THIN-CPRD dataset.^19 22^

Information on mothers and their children in the THIN-CPRD dataset can be linked using the family identification code, pregnancy codes, mother’s registered or estimated delivery date, child’s month of birth and gestational age at delivery. This is the optimal linkage method allowing identification of a large proportion of mother-child pairs.^23 24^ In addition, in both THIN and CPRD a large proportion of patients (about 30% and 60%, respectively) have linked hospital record data. Our estimates using THIN suggest that while the mode of delivery is accurately recorded in primary care (98% verified against hospital records), the recording is incomplete (the delivery mode is known for 55%–64% of children). The mode of delivery is well recorded in hospital records,^3^ therefore where linked hospital data are available, this will increase the sample of children with known mode of delivery where this is missing in primary care data.

To allow us to investigate more severe outcomes of interest requiring hospital admissions which are better recorded in secondary care, we will also create a mother-child linked database using anonymised Hospital Episode Statistics (HES) data collected for all NHS hospital admissions in England.^25^ This is a complex task requiring considerable expertise in record linkage, because in the UK there is no shared identifier to link maternal and child records in HES. It is, however, possible using deterministic and probabilistic linkage to attribute up to 98% of baby and mother secondary care records, as has been demonstrated in other large-scale studies of maternal and early life course research.^26 27^

We have a proposed linkage strategy which has already been validated by another recent study using matching algorithms based on HES data using organisation codes, admission dates, birth dates, general practice codes, sex, gestation and maternal age plus a number of other variables common to birth and maternity records. The database remains nationally representative for the main birth characteristics (such as gestational age, birth weight, sex and maternal age).^27^ The final output of this process will be a linked HES data set in which details of birth events and subsequent admissions of the children associated with these events can be elucidated.

HES alone, however, cannot be used to identify timing of prophylactic antibiotic administration and prophylactic antibiotics given. We will obtain the time point after which preincision antibiotic policy was introduced in each hospital from a national survey of maternity care providers in the UK. All maternity units undertaking CSs were included in the survey with a target response rate of 85%.

The exposure and outcome measures in the healthcare databases will be defined using the Read clinical code classification system used in primary care, and International Statistical Classification of Diseases and Related Health Problems 10th Revision (ICD-10) used for clinical diagnoses, the Office of Population Censuses and Surveys Classification of Surgical Operations and Procedures 4th revision (OPCS) for procedures and Healthcare Resource Groups (HRG) codes used in HES.

Recording of some variables in healthcare data, such as breastfeeding, is incomplete; we will therefore also investigate the trends in these variables by the mode of delivery using additional data sources such as the National Maternity Surveys.^28^

### Methods

We will compare rates of diagnosis of asthma and other outcomes of interest over time in children born by CS, comparing outcomes according to whether each mother received preincisional antibiotics.

In the primary analysis, we will estimate a probability that each mother received preincisional antibiotics according to year of birth, based on national policy uptake rates in the year of delivery for primary care data. For secondary care data, we will use the response from each hospital indicating the year of local policy implementation.

The major threat to validity in this observational comparison is not from case-mix confounders (indications for and incidence of CS have changed little over the study period); rather they relate to temporal changes in diagnosis and in the recording of outcomes and other exposures which impact on the number of cases identified in routine data. Patterns of diagnosis of childhood asthma, for example, have changed over time, in part driven by the revisions in the national asthma management guideline and the potentially conflicting compliance and prevalence issues faced in meeting specific indicators of the Quality and Outcomes Framework (QOF) introduced in 2004.^29 30^

An analysis reliant on adjustment for confounding factors is unlikely to succeed in controlling for these changes as it is unclear (1) what all the drivers of all these changes have been, (2) whether any covariates exist which accurately describe these changes without substantial missing data and (3) challenges in specification of a functional form for the relationship between these covariates and outcome.

In order to control for such temporal changes, we will use vaginally delivered (VD) children as a comparator, as this group will not have routinely received prophylactic antibiotics, but will have been subject to all the same temporal changes as those born by CS. Our study design will model the incidence of outcomes preintervention to predict the difference in disease incidence between babies born by CS (with antibiotics post cord-clamping) and VD (these are known to differ for some outcomes, such as asthma). From the period in which preincisional antibiotics are introduced, we will compare the observed incidence rate in CS children with a counterfactual incidence rate created by adding the VD-CS (postcord-clamping) difference to the observed incidence rate in VD children born postintervention. Subject to the assumption that the model of the difference between CS and VD rates is transferable across the time periods, differences between the observed and counterfactual CS event rates will be interpreted as likely to be caused by the change in practice.

### Model validity

To assess model validity, we will explore changes in the case-mix of covariates over time in relation to delivery mode. Both maternal and child characteristics will be explored. The maternal characteristics considered will be: age at childbirth, ethnicity, parity, smoking status, body mass index before pregnancy, area deprivation coding, long-term allergy-related health conditions (asthma, eczema, allergic rhinitis and conjunctivitis), pregnancy and labour complications (premature rupture of membranes, postpartum haemorrhage, manual placental removal/retained products of conception) and antibiotic prescribing during pregnancy. The characteristics of the child considered include: gestational age, sex, ethnicity, birth weight, breastfeeding status and antibiotic prescribing during the first 5 years.

### Estimates of sample size and statistical power

To obtain estimates of statistical power and to estimate the impact of misclassification on estimates of increase in risk with prophylactic antibiotics before CS, we simulated the study (1) based on our estimates using the THIN database regarding the number of children with linked maternal data and asthma diagnosis rates in each year group between birth and 5 years of age, in line with previously published figures,^31^ and (2) using HES data based on estimates of children with linked maternal data and rates of children newly hospitalised for asthma assuming a readmission rate during the follow-up period of 50% based on HES statistics.^25^

In each simulation, we created a dataset for the whole study, with 13 birth cohorts from 2006 to 2018, and follow-up included across the first 5 years of life (curtailed at the end of 2018) (table 2). Each birth was classified by mode of delivery, and for those delivered by CS, whether antibiotics were given before skin incision, generated randomly using a binomial random number generator using an underlying probability of exposure to preincision antibiotics during that year of birth.

**Table 2:** The number of births, years of follow-up and expected events in each simulation

	THIN-CPRD database	HES database
CS births	206 615	2 070 500
Postclamping antibiotics	111 508	1 115 670
Preincision antibiotics	95 107	954 830
VD births	570 774	5 973 100
Total births	777 389	8 043 600
CS person years of follow-up	792 265	8 661 832
Postclamping antibiotics	501 401	5 524 890
Preincision antibiotics	290 864	3 136 942
VD person years of follow-up	2 215 405	25 339 526
Total person years of follow-up	3 007 670	34 001 358
New events in children born by CS	7173	15 333
Postclamping antibiotics	5324	10 454
Preincision antibiotics	1849	4880
New events in children born by VD	20 378	44 906
Total events	27 551	60 240
Average event rate per 1000 person years	9.2	1.8

CPRD, Clinical Practice Research Datalink; CS, caesarean section; HES, Hospital Episode Statistics; THIN, The Health Improvement Network; VD, vaginal delivery.

Outcome events were randomly simulated according to the year-age event rates using a binomial random number generator, with increased rates in all those delivered by CS, and increased further in those who received antibiotics before skin incision. A risk ratio of 1.2 was used for increased risk of asthma with CS^32^ and then further increases with risk ratios from RR=1.10 to RR=1.20 (increasing in steps of 0.02) for the increase with antibiotics before skin incision rather than after cord-clamping.

Simulations were repeated 1000 times, and statistical power estimated by noting the proportion of simulations for which the lower limit of the 95% CI for the variable indicating whether antibiotics were given before skin incision was greater than a risk ratio of 1. We also recorded the estimates of the relative risk to assess attenuation bias created by misclassification.

The model which we fitted to analyse the simulation data included a trend term for the probability of receiving preincisional antibiotics with values 2006–2009=0, 2010=0.2, 2011=0.4, 2012=0.6, 2013=0.8, 2014–2018=1 with a zero value for those delivery vaginally (in the final analysis, we will use probabilities for each year obtained from the survey).

For the primary care data, we have 80% power of detecting a 16% relative increase in risk of asthma and over 90% power of detecting an 18% relative increase in risk, and being able to estimate them with a maximum of 15% underestimation from misclassification. For the HES admission data, we have over 80% power to detect a 10% relative increase in risk of asthma and 90% power to detect a 12% relative increase in risk with similar rates of underestimation due to misclassification.

The study will also be adequately powered to detect differences in the other primary outcome of interest (eczema) as incidence of clinician-diagnosed eczema is higher than asthma incidence in children in the UK.^33–35^

### Analysis

The primary and secondary outcomes will be analysed using a Poisson regression model to estimate the relative risk of developing each outcome with preincision compared with postcord clamping antibiotics. We will assess for overdispersion and if high, consider other models, such as a negative binomial. Appropriate considerations will be made to allow for the autocorrelation of data. We will look at the autocorrelation and partial autocorrelation plots to ensure any autocorrelation is accounted for. An adjustment for calendar time will be included in the model to allow for season effects. We will include terms for year, age and the interaction between them and mode of delivery (CS or vaginal). The key outcome parameter will be estimated by an additional term to identify those who receive preincision rather than postcord clamping antibiotics.

Rather than being described in dichotomous form, we will estimate the probability of preincision antibiotics using data from the national survey and known hospital policy. The estimated coefficient will provide an estimate of the change in policy, adjusting for misclassification.

#### Sensitivity analyses

Sensitivity to population changes:

- Analysis assessing the impact of the timing of the prophylactic antibiotic policy change, including comparison of analysis restricted to the years 2006–2010 (before the change in the NICE guideline) compared with years where over 50% of hospitals had introduced the policy.
- Analysis of the primary outcomes in the full HES dataset (including data for the hospitals that do not respond to the survey and therefore preclude us linking information about prophylactic antibiotic policy at hospital level) using the estimated probability of introduction of preincisional antibiotics according to calendar year, to investigate the consistency of findings.
- Analysis investigating the impact of the data recording quality (restricted to HES-linked records in THIN-CPRD database as the most accurate source of records for the mode of delivery).
- Exploratory sensitivity analysis employing the discordant sibling approach (restricting the analysis to women who gave birth by CS more than once during the study period including before and after the change in the prophylactic antibiotic policy compared with women who gave birth by VD more than once during the study period) to control further for family-related genetic and environmental factors.

Sensitivity to model changes:

- Analysis exploring whether the results are robust to the inclusion of a random effect for hospital.

Subgroup analyses:

- Exploratory subgroup analysis in HES mother-child linked database by prophylactic antibiotic type administered according to the individual hospital policies to investigate the potential impact of different antibiotics (cefuroxime alone, co-amoxiclav alone, cefuroxime+metronidazole) on child outcomes.
- Exploratory subgroup analysis by the type of CS (it is hypothesised that children delivered by elective CS have a higher likelihood of asthma and related outcomes and are more likely to be exposed to in-utero antibiotics for longer than children born to women having an emergency CS).

### Patient and public involvement (PPI)

We have involved the public throughout the development of this study. This has reconfirmed the importance of the research question, particularly: the importance of assuring the baby’s health as a main priority when deciding on delivery options; that uncertainty as to whether antibiotics given around the time of birth have an impact on children later in life should be resolved; that a robust study design is required to ensure the validity of the findings; a broad scope of important health and other outcomes which need to be considered; that the project needs to clearly communicate findings in terms of risks and benefits; that the findings regarding prophylactic antibiotics for CS should form part of the wider discussion regarding risks and benefits of medications in pregnancy.

Two lay parent representatives are members of our Project Management Group and an independent parent representative is a member of the Project Steering Group. We also held two PPI discussion groups with mothers and mothers-to-be in two different locations in the West Midlands. These women were from a range of backgrounds, including women from black and minority ethnic communities, a group often under-represented in research. The focus of the sessions was on exploring what women wanted to know about this research and particularly which health conditions in relation to this study were important to them. PPI helped us to confirm that we should look at a wide range of outcomes and also consider the severity of outcomes. In addition, a wider public consultation took place via a survey, a link to which was sent to the Royal College of Obstetricians & Gynaecologists (RCOG) Women’s Voices Involvement Panel and British Intrapartum Care Society (BICS) which includes lay members. Based on findings from the PPI workshops and the survey, we have added neurodevelopmental conditions as secondary/exploratory study outcomes.

Clear communication and publicising of key findings and messages are priorities of the study. Another PPI workshop is planned towards the end of the project to coproduce messages for dissemination via clinical networks, patient organisations and the media.

## Ethics and dissemination

Ethical approval for this study has been provided by the Ethical Review Committee of the University of Birmingham (ERN_17–1675). The THIN database was approved by the NHS South-East Multi-Centre Research Ethics Committee.^36^ Approval for the use of THIN and HES-linked data in this study was provided by the Independent Scientific Ethical Advisory Committee—Scientific Review Committee panel of the data provider, IQVIA (18THIN047). The CPRD has ethics approval for observational research using anonymised data from a National Research Ethics Committee.^37^ The use of CPRD for this study has been approved by the Independent Scientific Advisory Committee for MHRA Database Research (18_181AR2). The use of the HES database is exempt from NHS Research Ethics Committee approval because it involves the analysis of an existing dataset of non-identifiable data. Approval for the use of HES data was obtained as part of the standard NHS Digital data approval process.^38^ Health Research Authority (HRA) have confirmed that as the study involves linking anonymised patient data from established databases for our study only, HRA approval is not required.

The main aim of dissemination for this project is to ensure that parents-to-be and clinicians have clear information about the benefits and risks of preincision prophylactic antibiotics for CS based on the latest evidence to facilitate shared decision making. We will engage with the clinical and lay stakeholders throughout the project to benefit from the wider stakeholder input, to maximise the dissemination opportunities and to ensure that the research findings are communicated as widely as possible.

This will be achieved by: organising a further PPI workshop to produce a lay summary of the findings for wider dissemination, a dissemination event at the end of the project with lay, clinical stakeholders and professional organisations; dissemination to the clinical directors of maternity units; conference presentations, peer reviewed publications and dissemination via website and social media.

We will also maximise dissemination through: the Collaborations for Leadership in Applied Health Research and Care (CLAHRC) West Midlands (and its successor Applied Research Collaboration ARC) making use of their platform for dissemination; our strategic alliance, Birmingham Health Partners (BHP), which aligns three NHS trusts in the West Midlands area; the West Midlands Academic Health Science Network (AHSN) whose responsibility it is to adopt, diffuse and disseminate innovation in the NHS.

## Supplementary Material

Reviewer comments

Author's manuscript
