# Supplementary material for: Long-term impact of giving antibiotics before skin incision versus after cord clamping on children born by caesarean section: protocol for a longitudinal study based on UK electronic health records
Source: BMJ Open. 2019 Sep 26;9(9):e033013. doi: 10.1136/bmjopen-2019-033013 (PMC6773283; doi:10.1136/bmjopen-2019-033013)
Supplement: Author's manuscript [file bmjopen-2019-033013.draft_revisions.pdf]

BMJ Open

BMJ Open is committed to open peer review. As part of this commitment we make the peer review history of every article we publish publicly available.

When an article is published we post the peer reviewers' comments and the authors' responses online. We also post the versions of the paper that were used during peer review. These are the versions that the peer review comments apply to.

The versions of the paper that follow are the versions that were submitted during the peer review process. They are not the versions of record or the final published versions. They should not be cited or distributed as the published version of this manuscript.

BMJ Open is an open access journal and the full, final, typeset and author-corrected version of record of the manuscript is available on our site with no access controls, subscription charges or pay-per-view fees (http://bmjopen.bmj.com).

If you have any questions on BMJ Open's open peer review process please email info.bmjopen@bmj.com

BMJ Open

Long term impact of giving antibiotics before skin incision versus after cord clamping on children born by caesarean section: protocol for a longitudinal study based on UK electronic health records

Journal:	*BMJ Open*
Manuscript ID	bmjopen-2019-033013
Article Type:	Protocol
Date Submitted by the Author:	17-Jul-2019
Complete List of Authors:	Šumilo, Dana; University of Birmingham, Institute of Applied Health Research<br>Nirantharakumar, Krishnarajah; University of Birmingham, Institute of Applied Health Research & Midlands Health Data Research UK<br>Willis, Brian; University of Birmingham, Institute of Applied Health Research<br>Rudge, Gavin; University of Birmingham, Institute of Applied Health Research<br>Martin, James; University of Birmingham, Institute of Applied Health Research<br>Gokhale, Krishna; University of Birmingham, Institute of Applied Health Research<br>Thayakaran, Rasiah; University of Birmingham, Institute of Applied Health Research<br>Adderley, Nicola; Institute of Applied Health Research, University of Birmingham,<br>Chandan, Joht; University of Birmingham, Institute of Applied Health Research;<br>Okoth, Kelvin; University of Birmingham, Institute of Applied Health Research<br>Hewston, Ruth; Patient and Public contributor<br>Skrybant, Magdalena; Patient and Public contributor<br>Deeks, Jon; University of Birmingham, Institute of Applied Health Research<br>Brocklehurst, Peter; University of Birmingham, Institute of Applied Health Research
Keywords:	Caesarean section, antibiotic prophylaxis, child, Asthma < THORACIC MEDICINE, Eczema < DERMATOLOGY, immune system diseases

SCHOLARONE™
Manuscripts

Title

Long term impact of giving antibiotics before skin incision versus after cord clamping on children born by caesarean section: protocol for a longitudinal study based on UK electronic health records

Dana Šumilo[1*], Krishnarajah Nirantharakumar[1,2], Brian H Willis[1], Gavin Rudge[1], James Martin[1], Krishna Gokhale[1], Rasiah Thayakaran[1], Nicola J Adderley[1], Joht S Chandan[1], Kelvin Okoth[1], Ruth Hewston[3], Magdalena Skrybant[3], Jonathan J Deeks[1,4], Peter Brocklehurst[1]

1. Institute of Applied Health Research, University of Birmingham, Birmingham B15 2TT, UK
2. Midlands Health Data Research UK, University of Birmingham, Birmingham B15 2TT, UK
3. Patient and Public contributor
4. NIHR Birmingham Biomedical Research Centre, University Hospitals Birmingham NHS Foundation Trust and University of Birmingham, Birmingham B15 2TT, UK

*Corresponding author, e-mail: D.Sumilo@bham.ac.uk

Keywords

Caesarean section, antibiotic prophylaxis, child, asthma, eczema, immune system diseases

For peer review only - http://bmjopen.bmj.com/site/about/guidelines.xhtml

Abstract

Introduction

In the UK, about a quarter of women give birth by caesarean section and are offered prophylactic broad-spectrum antibiotics to reduce the risk of maternal postpartum infection. In 2011, national guidance was changed from recommending antibiotics after the umbilical cord was cut to giving antibiotics prior to skin incision based on evidence that earlier administration reduces maternal infectious morbidity. Although antibiotics cross the placenta, there are no known short-term harms to the baby. This study aims to address the research gap on longer term impact of these antibiotics on child health.

Methods and analysis

A controlled interrupted time series study will use anonymised mother-baby linked routine electronic health records for children born during 2006-2018 recorded in UK primary care (The Health Improvement Network, THIN, and Clinical Practice Research Datalink, CPRD) and secondary care (Hospital Episode Statistics, HES) databases. The primary outcomes of interest are asthma and eczema, two common allergy-related diseases in childhood. In-utero exposure to antibiotics immediately prior to caesarean section will be compared to no exposure when given after cord clamping. The risk of outcomes in children delivered by caesarean section will also be compared to a control cohort delivered vaginally to account for time effects. We will use all available data from THIN, CPRD and HES with estimated power of 80% and 90% to detect relative increase in risk of asthma of 16% and 18%, respectively at the 5% significance level.

Ethics and dissemination

Ethical approval has been obtained from the University of Birmingham Ethical Review Committee with scientific approvals obtained from the independent scientific advisory committees from the Medicines and Healthcare products Regulatory Agency for CPRD and the data provider, IQVIA for THIN. The results will be published in peer-reviewed journals, presented at national and international conferences, and disseminated to stakeholders.

Article Summary

Strengths and limitations of this study

- A large sample size including mother-child linked data from two nationally representative primary healthcare databases and a secondary healthcare database
- Investigation of a broad range of relevant child outcomes including severity
- Investigation of maternal outcomes using real wold evidence to confirm findings reported in randomised controlled trials
- Use of a comparison group of vaginally delivered children to effectively control for changes in diagnosis, recording and exposures over time
- Timing of prophylactic antibiotic administration is not recorded in routine healthcare data, therefore analysis is based on the estimated proportion of hospitals with the pre-incision antibiotic policy in each year during the study period

For peer review only - http://bmjopen.bmj.com/site/about/guidelines.xhtml

Introduction

Births by caesarean section (CS) account for over 20% of births globally and are increasing (1). Over one in four babies in the UK are born by CS (2-5). CS is a surgical procedure and women undergoing CS are at increased risk of developing infections after giving birth which can be prevented by prophylactic antibiotics. Before 2011, the national guidance advised administering intravenous prophylactic antibiotics for women undergoing CS after the baby's cord had been clamped to prevent exposing the baby to antibiotics. In 2011, the guidance was changed to recommend giving antibiotics to women undergoing CS prior to skin incision. This was based on evidence that earlier administration reduces maternal infectious morbidity (6). The current Cochrane review summarises data from 10 randomised trials (5,041 women) which showed a near halving of risk of all postpartum maternal infection (43%, 95% confidence interval (CI) 28-55%), endometritis (46%, CI 21-64%), and wound infection (41%, CI 19-56%) compared with giving antibiotics after clamping the baby's umbilical cord (7). Most postpartum maternal infections, however, are mild and respond well to treatment (8).

Preoperative prophylactic antibiotics rapidly cross the placenta exposing babies to high dose broad spectrum antibiotics around the time of birth (9). Although no short-term harms to the baby have been reported (6), intrapartum antibiotics have been shown to alter the gut microbiota of newborns (10). There is growing evidence that the composition of gut microbes in infants plays a role in their immune system development including response to different antigens and inflammation, and is associated with susceptibility to asthma, allergies and other immune-related diseases later in life (11-18). There is a paucity of research regarding the longer term effect of pre-incision prophylactic antibiotics for CS on child health.

Aim

The overall aim of this research study is to investigate whether the change in the guidance from recommending prophylactic antibiotics after cord clamping to pre-incision antibiotics has had any effect on the incidence of allergic and other related health conditions in children born by CS in the UK. This study will provide evidence on long term impacts of CS preoperative prophylactic antibiotics to inform current guidance regarding the timing of administration of these antibiotics. It will either reinforce the current recommendation or, if negative impacts on child health are observed, will enable assessment of the magnitude of the risks against the benefits of reduced maternal morbidity.

Objectives

The primary objective of the study is to investigate whether pre-incisional in-utero exposure to antibiotics immediately prior to birth (Intervention) compared to no pre-incisional antibiotic exposure (Comparator) increases the risk of 1) asthma and 2) eczema (Outcomes) in children born by CS (Population). The relationship between antibiotic exposure and asthma and eczema severity (defined based on prescribing information and hospital admission data) will also be explored.

For peer review only - http://bmjopen.bmj.com/site/about/guidelines.xhtml

Secondary objectives:

1. Investigating the effect of pre-incision prophylactic antibiotics in children born by CS on: a) other allergic and allergy-related diseases; b) autoimmune diseases; c) infections and inflammation; d) other immune system-related conditions; e) neurodevelopmental conditions; f) less specific measures of child health (colic and failure to thrive).

2. Investigating the effect of pre-incision prophylactic antibiotics in children born by CS on health service utilisation (overall consultation frequency in primary care and hospital admissions).

3. Investigating if the effects of a reduction in post-partum maternal infectious morbidity shown in randomised controlled trials outside the UK can be replicated in the UK using routine healthcare data.

Methods and analysis

Study design

To address the primary objective and secondary objectives 1 and 2, a controlled interrupted time series study will be undertaken using a cohort of women and their children born between 2006 and 2018 in the UK who are included in two routine primary care databases, The Health Improvement Network (THIN) or Clinical Practice Research Datalink (CPRD), and the secondary care Hospital Episode Statistics (HES) database.

Target population

Children born by CS and exposed, in utero, to antibiotics immediately prior to birth will be compared with children born by CS and not exposed, in utero, to antibiotics immediately prior to birth. Children born vaginally during the same time period will be included as a control group.

Eligibility criteria

All liveborn children for whom the birth year is between 2006 and 2018 will be included; the child and their mother's healthcare record can be linked in primary care (THIN or CPRD) or secondary care (HES) databases; the mode of delivery, CS or vaginal delivery (VD), can be identified based on recording in primary care (THIN, CPRD) and/or secondary care (HES).

Exclusion criteria

Children with missing delivery information will be excluded. In case of multiple births (e.g. twins), one of the children will be randomly selected for inclusion to ensure independence of observations.

Study outcomes

The primary outcomes for the study are the incidence of 1) asthma and 2) eczema. The main analysis for primary outcomes will be done separately in the primary care dataset and the

For peer review only - http://bmjopen.bmj.com/site/about/guidelines.xhtml

secondary care (HES) dataset (the latter including only hospitals for which the year of antibiotic prescribing policy change is known).

Secondary outcomes are other allergic and allergy-related diseases, autoimmune diseases, infections and inflammation, other immune system-related conditions, neurodevelopmental conditions, less specific measures of child health, healthcare utilisation, and maternal postpartum infectious morbidity (Table 1).

Table 1. The list of secondary outcomes.

Outcome	Corresponding secondary objective	Datasets analysed	
		Primary care	Secondary care
**Health conditions and symptoms in children**	1.		
Other allergic and allergy-related conditions:	1.a		
• food allergy/intolerance		x	
• allergic rhinitis and conjunctivitis		x	
• >1 allergy related disease (asthma, eczema, food allergy/intolerance, allergic rhinitis and conjunctivitis)		x	
• penicillin allergy*		x	
• anaphylaxis*		x	x
• high risk of anaphylactic reaction (prescribing of automatic injection devices containing adrenaline)*		x	
Autoimmune diseases:	1.b		
• type 1 diabetes*		x	x
• coeliac disease*		x	x
• juvenile idiopathic arthritis*		x	x
• scleroderma/systemic sclerosis*†		x	x
• inflammatory myopathies*†		x	x
• systemic lupus erythematosus (SLE)*†		x	x
• autoimmune (idiopathic) thrombocytopenic purpura (ITP)*		x	x
• juvenile pernicious (megaloblastic) anaemia*		x	x
• childhood vitiligo*†		x	
Infections and inflammation:	1.c		
• neonatal sepsis (early and late onset)			x
• other sepsis*			x
• wheeze		x	
• upper respiratory tract infections*		x	
• lower respiratory tract infections*		x	x
• bronchiolitis*		x	x
• gastroenteritis*		x	x
• inflammatory bowel disease†		x	x
• urinary tract infections*		x	x
• antibiotic prescribing*		x	
Other immune system-related conditions:	1.d		
• necrotising enterocolitis			x
• leukaemia*†		x	x

For peer review only - http://bmjopen.bmj.com/site/about/guidelines.xhtml

Neurodevelopmental conditions:	1.e		
• cerebral palsy		x	
• autism spectrum disorder*		x	
• attention deficit hyperactivity disorder (ADHD)*		x	
Less specific measures of child health:	1.f		
• colic*		x	
• failure to thrive*		x	
**Healthcare utilisation in children**	2.		
• primary care consultations*		x	
• hospital admissions*			x
**Maternal outcomes** (six weeks post-partum)	3.		
• composite infectious morbidity (wound infection, endometritis/endomyometritis, pelvic abscess, maternal sepsis, death attributed to infection)		x	x
• endometritis/endomyometritis		x	x
• wound infection		x	x
• urinary tract infection/cystitis/pyelonephritis		x	x
• sepsis		x	x
• pelvic abscess		x	x
• maternal death (if infection related)*†			x
• antibiotic prescribing*		x	
• length of hospital stay*			x

* Exploratory outcome due to insufficient evidence base, including lack of longitudinal studies investigating the association between microbiota/early antibiotic exposure and outcome of interest; †Tabulation if the outcome is very rare.

Data sources

To maximise the sample size, we will combine two UK-wide primary care research databases, THIN and CPRD, containing anonymised patient records of over 10% of the UK patient population (19). Both databases are broadly generalisable to the UK population in terms of demographics and medical condition prevalence (20, 21). There is overlap between the databases at general practice level, with THIN and CPRD containing 37% and 46% unique practices, respectively. The databases do not use the same identifiers for patients or practices, but the overlapping practices can be identified reliably using patient registration, demographic and medical record information, and the duplicates removed to create a combined THIN-CPRD dataset (19, 22).

Information on mothers and their children in the THIN-CPRD dataset can be linked using the family identification code, pregnancy codes, mother's registered or estimated delivery date, child's month of birth, and gestational age at delivery. This is the optimal linkage method allowing identification of a large proportion of mother-child pairs (23, 24). In addition, in both THIN and CPRD a large proportion of patients (about 30% and 60%, respectively) have linked hospital record data. Our estimates using THIN suggest that whilst the mode of delivery is accurately recorded in primary care (98% verified against hospital records), the recording is incomplete (the delivery mode is known for 55-64% of children). The mode of delivery is well recorded in hospital records (3), therefore where linked hospital data are

For peer review only

available, this will increase the sample of children with known mode of delivery where this is missing in primary care data.

To allow us to investigate more severe outcomes of interest requiring hospital admissions which are better recorded in secondary care, we will also create a mother-child linked database using anonymised Hospital Episode Statistics (HES) data collected for all NHS hospital admissions in England (25). This is a complex task requiring considerable expertise in record linkage, because in the UK there is no shared identifier to link maternal and child records in HES. It is, however, possible using deterministic and probabilistic linkage to attribute up to 98% of baby and mother secondary care records, as has been demonstrated in other large-scale studies of maternal and early life course research (26, 27).

We have a proposed linkage strategy which has already been validated by another recent study using matching algorithms based on HES data using organisation codes, admission dates, birth dates, GP practice codes, sex, gestation and maternal age plus a number of other variables common to birth and maternity records. The database remains nationally representative for the main birth characteristics (such as gestational age, birthweight, sex and maternal age) (27). The final output of this process will be a linked HES data set in which details of birth events and subsequent admissions of the children associated with these events can be elucidated.

HES alone, however, cannot be used to identify timing of prophylactic antibiotic administration and prophylactic antibiotics given. We will obtain the time point after which pre-incision antibiotic policy was introduced in each hospital from a national survey of maternity care providers in the UK. All maternity units undertaking caesarean sections were included in the survey with a target response rate of 85%.

The exposure and outcome measures in the healthcare databases will be defined using the Read clinical code classification system used in primary care, and International Statistical Classification of Diseases and Related Health Problems 10th Revision (ICD-10) used for clinical diagnoses, the Office of Population Censuses and Surveys Classification of Surgical Operations and Procedures 4th revision (OPCS) for procedures and Healthcare Resource Groups (HRG) codes used in HES.

Recording of some variables in healthcare data, such as breastfeeding, is incomplete; we will therefore also investigate the trends in these variables by the mode of delivery using additional data sources such as the National Maternity Surveys (28).

Methods

We will compare rates of diagnosis of asthma and other outcomes of interest over time in children born by CS, comparing outcomes according to whether each mother received pre-incisional antibiotics.

In the primary analysis we will estimate a probability that each mother received pre-incisional antibiotics according to year of birth, based on national policy uptake rates in the year of delivery for primary care data. For secondary care data, we will use the response from each hospital indicating the year of local policy implementation.

For peer review only - http://bmjopen.bmj.com/site/about/guidelines.xhtml

The major threat to validity in this observational comparison is not from case-mix confounders (indications for and incidence of CS have changed little over the study period); rather they relate to temporal changes in diagnosis and in the recording of outcomes and other exposures which impact on the number of cases identified in routine data. Patterns of diagnosis of childhood asthma, for example, have changed over time, in part driven by the revisions in the national asthma management guideline and the potentially conflicting compliance and prevalence issues faced in meeting specific indicators of the Quality and Outcomes Framework (QOF) introduced in 2004 (29, 30).

An analysis reliant on adjustment for confounding factors is unlikely to succeed in controlling for these changes as it is unclear (a) what all the drivers of all these changes have been, (b) whether any covariates exist which accurately describe these changes without substantial missing data, and (c) challenges in specification of a functional form for the relationship between these covariates and outcome.

In order to control for such temporal changes, we will use vaginally delivered (VD) children as a comparator, as this group will not have routinely received prophylactic antibiotics, but will have been subject to all the same temporal changes as those born by CS. Our study design will model the incidence of outcomes pre-intervention to predict the difference in disease incidence between babies born by CS (with antibiotics post cord-clamping) and VD (these are known to differ for some outcomes, such as asthma). From the period in which pre-incisional antibiotics are introduced, we will compare the observed incidence rate in CS children with a counterfactual incidence rate created by adding the VD-CS (post cord-clamping) difference to the observed incidence rate in VD children born post-intervention. Subject to the assumption that the model of the difference between CS and VD rates is transferable across the time periods, differences between the observed and counterfactual CS event rates will be interpreted as likely to be caused by the change in practice.

Model validity

To assess model validity, we will explore changes in the case-mix of covariates over time in relation to delivery mode. Both maternal and child characteristics will be explored. The maternal characteristics considered will be: age at childbirth, ethnicity, parity, smoking status, body mass index (BMI) before pregnancy, area deprivation coding, long term allergy-related health conditions (asthma, eczema, allergic rhinitis and conjunctivitis), pregnancy and labour complications (premature rupture of membranes, post-partum haemorrhage, manual placental removal/retained products of conception), and antibiotic prescribing during pregnancy. The characteristics of the child considered include: gestational age, sex, ethnicity, birthweight, breastfeeding status, and antibiotic prescribing during the first five years.

Estimates of sample size and statistical power

To obtain estimates of statistical power, and to estimate the impact of misclassification on estimates of increase in risk with prophylactic antibiotics before CS, we simulated the study (a) based on our estimates using the THIN database regarding the number of children with linked maternal data and asthma diagnosis rates in each year group between birth and 5

For peer review only - http://bmjopen.bmj.com/site/about/guidelines.xhtml

years of age, in line with previously published figures (31), and (b) using HES data based on estimates of children with linked maternal data and rates of children newly hospitalised for asthma assuming a readmission rate during the follow up period of 50% based on HES statistics (25).

In each simulation we created a dataset for the whole study, with 13 birth cohorts from 2006 to 2018, and follow-up included across the first 5 years of life (curtailed at the end of 2018) (Table 2). Each birth was classified by mode of delivery, and for those delivered by CS, whether antibiotics were given before skin incision, generated randomly using a binomial random number generator using an underlying probability of exposure to pre-incision antibiotics during that year of birth.

Table 2. The number of births, years of follow-up and expected events in each simulation.

	THIN-CPRD database	HES database
**CS births**	206,615	2,070,500
Post-clamping antibiotics	111,508	1,115,670
Pre-incision antibiotics	95,107	954,830
**VD births**	570,774	5,973,100
**Total births**	777,389	8,043,600
**CS person years of follow-up**	792,265	8,661,832
Post-clamping antibiotics	501,401	5,524,890
Pre-incision antibiotics	290,864	3,136,942
**VD person years of follow-up**	2,215,405	25,339,526
**Total person years of follow-up**	3,007,670	34,001,358
**New events in children born by CS**	7,173	15,333
Post-clamping antibiotics	5,324	10,454
Pre-incision antibiotics	1,849	4,880
**New events in children born by VD**	20,378	44,906
**Total events**	27,551	60,240
**Average event rate per 1000 person years**	9.2	1.8

Outcome events were randomly simulated according to the year-age event rates using a binomial random number generator, with increased rates in all those delivered by CS, and increased further in those who received antibiotics before skin incision. A risk ratio of 1.2 was used for increased risk of asthma with CS (32), and then further increases with risk ratios from RR=1.10 to RR=1.20 (increasing in steps of 0.02) for the increase with antibiotics before skin incision rather than after cord-clamping.

Simulations were repeated 1000 times, and statistical power estimated by noting the proportion of simulations for which the lower limit of the 95% confidence interval for the variable indicating whether antibiotics were given before skin incision was greater than a risk ratio of 1. We also recorded the estimates of the relative risk to assess attenuation bias created by misclassification.

For peer review only - http://bmjopen.bmj.com/site/about/guidelines.xhtml

The model which we fitted to analyse the simulation data included a trend term for the probability of receiving pre-incisional antibiotics with values 2006-2009=0, 2010=0.2, 2011=0.4, 2012=0.6, 2013=0.8, 2014-2018=1 with a zero value for those delivery vaginally (in the final analysis we will utilise probabilities for each year obtained from the survey).

For the primary care data we have 80% power of detecting a 16% relative increase in risk of asthma and over 90% power of detecting an 18% relative increase in risk, and being able to estimate them with a maximum of 15% underestimation from misclassification. For the HES admission data, we have over 80% power to detect a 10% relative increase in risk of asthma and 90% power to detect a 12% relative increase in risk with similar rates of underestimation due to misclassification.

The study will also be adequately powered to detect differences in the other primary outcome of interest (eczema) as incidence of GP diagnosed eczema is higher than asthma incidence in children in the UK (33-35).

Analysis

The primary and secondary outcomes will be analysed using a Poisson regression model to estimate the relative risk of developing each outcome with pre-incision compared to post-cord clamping antibiotics. We will assess for over-dispersion and if high, consider other models, such as a negative binomial. Appropriate considerations will be made to allow for the auto-correlation of data. We will look at the autocorrelation and partial autocorrelation plots to ensure any autocorrelation is accounted for. An adjustment for calendar time will be included in the model to allow for season effects. We will include terms for year, age, and the interaction between them and mode of delivery (CS or vaginal). The key outcome parameter will be estimated by an additional term to identify those who receive pre-incision rather than post-cord clamping antibiotics.

Rather than being described in dichotomous form, we will estimate the probability of pre-incision antibiotics using data from the national survey and known hospital policy. The estimated coefficient will provide an estimate of the change in policy, adjusting for misclassification.

Sensitivity analyses

Sensitivity to population changes:

- Analysis assessing the impact of the timing of the prophylactic antibiotic policy change, including comparison of analysis restricted to the years 2006-2010 (before the change in the NICE guideline) compared to years where over 50% of hospitals had introduced the policy;

- Analysis of the primary outcomes in the full HES dataset (including data for the hospitals that do not respond to the survey and therefore preclude us linking information about prophylactic antibiotic policy at hospital level) using the estimated probability of introduction of pre-incisional antibiotics according to calendar year, to investigate the consistency of findings;

For peer review only - http://bmjopen.bmj.com/site/about/guidelines.xhtml

- Analysis investigating the impact of the data recording quality (restricted to HES-linked records in THIN-CPRD database as the most accurate source of records for the mode of delivery);
- Exploratory sensitivity analysis employing the discordant sibling approach (restricting the analysis to women who gave birth by CS more than once during the study period including before and after the change in the prophylactic antibiotic policy compared to women who gave birth by VD more than once during the study period) to control further for family-related genetic and environmental factors.

Sensitivity to model changes:

- Analysis exploring whether the results are robust to the inclusion of a random effect for hospital.

Subgroup analyses:

- Exploratory subgroup analysis in HES mother-child linked database by prophylactic antibiotic type administered according to the individual hospital policies to investigate the potential impact of different antibiotics (cefuroxime alone, co-amoxiclav alone, cefuroxime + metronidazole) on child outcomes;
- Exploratory subgroup analysis by the type of CS (it is hypothesised that children delivered by elective CS have a higher likelihood of asthma and related outcomes and are more likely to be exposed to in-utero antibiotics for longer than children born to women having an emergency CS).

Patient and public involvement (PPI)

We have involved the public throughout the development of this study. This has reconfirmed the importance of the research question, particularly: the importance of assuring the baby's health as a main priority when deciding on delivery options; that uncertainty as to whether antibiotics given around the time of birth have an impact on children later in life should be resolved; that a robust study design is required to ensure the validity of the findings; a broad scope of important health and other outcomes which need to be considered; that the project needs to clearly communicate findings in terms of risks and benefits; that the findings regarding prophylactic antibiotics for CS should form part of the wider discussion regarding risks and benefits of medications in pregnancy.

Two lay parent representatives are members of our Project Management Group and an independent parent representative is a member of the Project Steering Group. We also held two PPI discussion groups with mothers and mothers-to-be in two different locations in the West Midlands. These women were from a range of backgrounds, including women from black and minority ethnic communities, a group often under-represented in research. The focus of the sessions was on exploring what women wanted to know about this research and particularly which health conditions in relation to this study were important to them. PPI helped us to confirm that we should look at a wide range of outcomes and also consider the severity of outcomes. In addition, a wider public consultation took place via a survey, a

For peer review only - http://bmjopen.bmj.com/site/about/guidelines.xhtml

For peer review only

link to which was sent to the Royal College of Obstetricians & Gynaecologists (RCOG) Women's Voices Involvement Panel and British Intrapartum Care Society (BICS) which includes lay members. Based on findings from the PPI workshops and the survey, we have added neurodevelopmental conditions as secondary/exploratory study outcomes.

Clear communication and publicising of key findings and messages are priorities of the study. Another PPI workshop is planned towards the end of the project to co-produce messages for dissemination via clinical networks, patient organisations and the media.

Ethics and dissemination

Ethical approval for this study has been provided by the Ethical Review Committee of the University of Birmingham (ERN_17-1675). The THIN database was approved by the NHS South-East Multi-Centre Research Ethics Committee (36). Approval for the use of THIN and HES-linked data in this study was provided by the Independent Scientific Ethical Advisory Committee - Scientific Review Committee panel of the data provider, IQVIA (18THIN047). The CPRD has ethics approval for observational research using anonymised data from a National Research Ethics Committee (37). The use of CPRD for this study has been approved by the Independent Scientific Advisory Committee for MHRA Database Research (18_181AR2). The use of the HES database is exempt from NHS Research Ethics Committee approval because it involves the analysis of an existing dataset of non-identifiable data. Approval for the use of HES data was obtained as part of the standard NHS Digital data approval process (38). Health Research Authority (HRA) have confirmed that as the study involves linking anonymised patient data from established databases for our study only, HRA approval is not required.

The main aim of dissemination for this project is to ensure that parents-to-be and clinicians have clear information about the benefits and risks of pre-incision prophylactic antibiotics for CS based on the latest evidence to facilitate shared decision making. We will engage with the clinical and lay stakeholders throughout the project to benefit from the wider stakeholder input, to maximise the dissemination opportunities, and to ensure that the research findings are communicated as widely as possible.

This will be achieved by: organising a further PPI workshop to produce a lay summary of the findings for wider dissemination, a dissemination event at the end of the project with lay, clinical stakeholders and professional organisations; dissemination to the clinical directors of maternity units; conference presentations, peer reviewed publications, and dissemination via website and social media.

We will also maximise dissemination through: the Collaborations for Leadership in Applied Health Research and Care (CLAHRC) West Midlands (and its successor Applied Research Collaboration ARC) making use of their platform for dissemination; our strategic alliance, Birmingham Health Partners (BHP), which aligns three NHS trusts in the West Midlands area; the West Midlands Academic Health Science Network (AHSN) whose responsibility it is to adopt, diffuse and disseminate innovation in the NHS.

For peer review only - http://bmjopen.bmj.com/site/about/guidelines.xhtml

Authors' contributions

DS, PB, JD, KN, BW, MS and RH participated in the conception and the initial design of the study, with further substantial contributions from GR, KG, NA, JM, RT, JS and KO. All authors participated in drafting the protocol and have approved the final version.

Acknowledgements

This study will be based in part on data from the Clinical Practice Research Datalink obtained under licence from the UK Medicines and Healthcare products Regulatory Agency and The Health Improvement Network obtained under licence from IQVIA. The data is provided by patients and collected by the NHS as part of their care and support. The interpretation and conclusions contained in this study will be those of the authors alone.

The OPCS Classification of Interventions and Procedures, codes, terms and text is Crown copyright (2016) published by Health and Social Care Information Centre, also known as NHS Digital and licenced under the Open Government Licence available at www.nationalarchives.gov.uk/doc/open-government-licence/open-government-licence.htm. Hospital Episode Statistics reused with the permission of the NHS Digital, under a data sharing agreement.

Funding

This study is funded by the National Institute for Health Research (NIHR) Health Technology Assessment (HTA) programme (16/150/01). The views expressed are those of the author(s) and not necessarily those of the NIHR or the Department of Health and Social Care.

Competing interests

None declared.

Data availability

Data for this study will be derived from THIN and CPRD primary care and linked data obtained under license from IQVIA World Publications Ltd and UK Medicines and Healthcare products Regulatory Agency. A secondary care database will be derived from HES data obtained under licence from the Health and Social Care Information Centre (NHS Digital). Data for similar cohorts can be requested from IQVIA World Publications Ltd, the UK Medicines and Healthcare Products Regulatory Agency and NHS Digital subject to protocol approval and license agreements.

References

1. Boerma T, Ronsmans C, Melesse DY, Barros AJD, Barros FC, Juan L, et al. Global epidemiology of use of and disparities in caesarean sections. Lancet. 2018;392(10155):1341-8.
2. Information Services Division Scotland. Births in Scotish Hospitals [Available from: https://www.isdscotland.org/Health-Topics/Maternity-and-Births/Births/].
3. NHS Digital. NHS Maternity Statistics, England [Available from: https://digital.nhs.uk/data-and-information/publications/statistical/nhs-maternity-statistics].

For peer review only - http://bmjopen.bmj.com/site/about/guidelines.xhtml

4. Public Health Agency of Northern Ireland. Children's Health in Northern Ireland [Available from: https://www.publichealth.hscni.net/directorates/operations/statistics].
5. Welsh Government. Maternity statistics [Available from: https://gov.wales/maternity-statistics].
6. National Collaborating Centre for Women’s and Children’s Health. Caesarean section. NICE Clinical guideline CG132. National Institute for Health and Care Excellence; 2011.
7. Mackeen AD, Packard RE, Ota E, Berghella V, Baxter JK. Timing of intravenous prophylactic antibiotics for preventing postpartum infectious morbidity in women undergoing cesarean delivery. The Cochrane database of systematic reviews. 2014(12):CD009516.
8. Bailey SR, Field N, Townsend CL, Rodger AJ, Brocklehurst P. Antibiotic prophylaxis for women undergoing caesarean section and infant health. BJOG : an international journal of obstetrics and gynaecology. 2016;123(6):875-6.
9. Sutton AL, Acosta EP, Larson KB, Kerstner-Wood CD, Tita AT, Biggio JR. Perinatal pharmacokinetics of azithromycin for cesarean prophylaxis. Am J Obstet Gynecol. 2015;212(6):812 e1-6.
10. Azad MB, Konya T, Persaud RR, Guttman DS, Chari RS, Field CJ, et al. Impact of maternal intrapartum antibiotics, method of birth and breastfeeding on gut microbiota during the first year of life: a prospective cohort study. BJOG : an international journal of obstetrics and gynaecology. 2016;123(6):983-93.
11. Lawley TD, Walker AW. Intestinal colonization resistance. Immunology. 2013;138(1):1-11.
12. Murgas Torrazza R, Neu J. The developing intestinal microbiome and its relationship to health and disease in the neonate. Journal of perinatology : official journal of the California Perinatal Association. 2011;31 Suppl 1:S29-34.
13. Honda K, Littman DR. The microbiome in infectious disease and inflammation. Annu Rev Immunol. 2012;30:759-95.
14. Penders J, Thijs C, van den Brandt PA, Kummeling I, Snijders B, Stelma F, et al. Gut microbiota composition and development of atopic manifestations in infancy: the KOALA Birth Cohort Study. Gut. 2007;56(5):661-7.
15. Tamburini S, Shen N, Wu HC, Clemente JC. The microbiome in early life: implications for health outcomes. Nat Med. 2016;22(7):713-22.
16. Bisgaard H, Bonnelykke K, Stokholm J. Immune-mediated diseases and microbial exposure in early life. Clin Exp Allergy. 2014;44(4):475-81.
17. Neu J, Rushing J. Cesarean versus vaginal delivery: long-term infant outcomes and the hygiene hypothesis. Clin Perinatol. 2011;38(2):321-31.
18. Jandhyala SM, Talukdar R, Subramanyam C, Vuyyuru H, Sasikala M, Nageshwar Reddy D. Role of the normal gut microbiota. World J Gastroenterol. 2015;21(29):8787-803.
19. Petersen I, McCrea R, Sammon C, Osborn D, Evans S, Cowen P, et al. Risks and benefits ofpsychotropic medication in pregnancy: cohort studies based on UK electronic primary care health records. Health Technology Assessment. 2016;20(23).
20. Blak BT, Thompson M, Dattani H, Bourke A. Generalisability of The Health Improvement Network (THIN) database: demographics, chronic disease prevalence and mortality rates. Inform Prim Care. 2011;19(4):251-5.
21. Williams T, van Staa T, Puri S, Eaton S. Recent advances in the utility and use of the General Practice Research Database as an example of a UK Primary Care Data resource. Ther Adv Drug Saf. 2012;3(2):89-99.
22. Cai B, Xu W, Bortnichak E, Watson DJ. An algorithm to identify medical practices common to both the General Practice Research Database and The Health Improvement Network database. Pharmacoepidemiol Drug Saf. 2012;21(7):770-4.
23. Cea-Soriano L, Rodriguez LAG, Cantero OF, Hernandez-Diaz S. Challenges of using primary care electronic medical records in the UK to study medications in pregnancy. Pharmacoepidem Dr S. 2013;22(9):977-85.

For peer review only

For peer review only - http://bmjopen.bmj.com/site/about/guidelines.xhtml

24. Charlton R, Snowball J, Sammon C, de Vries C. The Clinical Practice Research Datalink for drug safety in pregnancy research: an overview. Therapie. 2014;69(1):83-9.
25. NHS Digital. Hospital Episode Statistics. [Available from: http://content.digital.nhs.uk/hes].
26. Davidson R, Roberts SE, Wotton CJ, Goldacre MJ. Influence of maternal and perinatal factors on subsequent hospitalisation for asthma in children: evidence from the Oxford record linkage study. BMC Pulm Med. 2010;10:14.
27. Harron K, Gilbert R, Cromwell D, van der Meulen J. Linking Data for Mothers and Babies in De-Identified Electronic Health Data. PLoS One. 2016;11(10):e0164667.
28. National Perinatal Epidemiology Unit. National maternity surveys. [Available from: https://www.npeu.ox.ac.uk/maternity-surveys].
29. British Thoracic Society. British guideline on the management of asthma. Scottish Intercollegiate Guidelines Network 2016.
30. NHS Digital. Quality and Outcomes Framework [Available from: http://content.digital.nhs.uk/qof].
31. British Lung Foundation. Asthma statistics [Available from: https://statistics.blf.org.uk/asthma].
32. Thavagnanam S, Fleming J, Bromley A, Shields MD, Cardwell CR. A meta-analysis of the association between Caesarean section and childhood asthma. Clin Exp Allergy. 2008;38(4):629-33.
33. Pols DH, Wartna JB, Moed H, van Alphen EI, Bohnen AM, Bindels PJ. Atopic dermatitis, asthma and allergic rhinitis in general practice and the open population: a systematic review. Scand J Prim Health Care. 2016;34(2):143-50.
34. Punekar YS, Sheikh A. Establishing the incidence and prevalence of clinician-diagnosed allergic conditions in children and adolescents using routinely collected data from general practices. Clin Exp Allergy. 2009;39(8):1209-16.
35. Ban L, Langan SM, Abuabara K, Thomas KS, Abdul Sultan A, Sach T, et al. Incidence and sociodemographic characteristics of eczema diagnosis in children: A cohort study. J Allergy Clin Immunol. 2018;141(5):1927-9 e8.
36. NHS Health Research Authority. The Health Improvement Network (THIN) database [Available from: https://www.hra.nhs.uk/planning-and-improving-research/application-summaries/research-summaries/the-health-improvement-network-thin-database/].
37. Clinical Practice Research Datalink. Safeguarding patient data [Available from: https://www.cprd.com/safeguarding-patient-data].
38. NHS Digital. Obtaining data from NHS Digital from health research - a guide for researchers. 2016.

For peer review only

For peer review only - http://bmjopen.bmj.com/site/about/guidelines.xhtml

STROBE Statement—checklist of items that should be included in reports of observational studies

	Item No	Recommendation	Page No
**Title and abstract**	1	(*a*) Indicate the study's design with a commonly used term in the title or the abstract	2
		(*b*) Provide in the abstract an informative and balanced summary of what was done and what was found	2
**Introduction**			
Background/rationale	2	Explain the scientific background and rationale for the investigation being reported	3
Objectives	3	State specific objectives, including any prespecified hypotheses	3-4
**Methods**			
Study design	4	Present key elements of study design early in the paper	4;7-8
Setting	5	Describe the setting, locations, and relevant dates, including periods of recruitment, exposure, follow-up, and data collection	4;9
Participants	6	(*a*) *Cohort study*—Give the eligibility criteria, and the sources and methods of selection of participants. Describe methods of follow-up<br>*Case-control study*—Give the eligibility criteria, and the sources and methods of case ascertainment and control selection. Give the rationale for the choice of cases and controls<br>*Cross-sectional study*—Give the eligibility criteria, and the sources and methods of selection of participants	4, 6-7
		(*b*) *Cohort study*—For matched studies, give matching criteria and number of exposed and unexposed<br>*Case-control study*—For matched studies, give matching criteria and the number of controls per case	
Variables	7	Clearly define all outcomes, exposures, predictors, potential confounders, and effect modifiers. Give diagnostic criteria, if applicable	4-6; 8
Data sources/ measurement	8*	For each variable of interest, give sources of data and details of methods of assessment (measurement). Describe comparability of assessment methods if there is more than one group	5-7
Bias	9	Describe any efforts to address potential sources of bias	8
Study size	10	Explain how the study size was arrived at	6-7; 9
Quantitative variables	11	Explain how quantitative variables were handled in the analyses. If applicable, describe which groupings were chosen and why	10
Statistical methods	12	(*a*) Describe all statistical methods, including those used to control for confounding	7-8; 10
		(*b*) Describe any methods used to examine subgroups and interactions	11
		(*c*) Explain how missing data were addressed	11
		(*d*) *Cohort study*—If applicable, explain how loss to follow-up was addressed<br>*Case-control study*—If applicable, explain how matching of cases and controls was addressed<br>*Cross-sectional study*—If applicable, describe analytical methods taking account of sampling strategy	9-10
		(*e*) Describe any sensitivity analyses	11

Continued on next page

For peer review only - http://bmjopen.bmj.com/site/about/guidelines.xhtml

**Results**			
Participants	13*	(a) Report numbers of individuals at each stage of study—eg numbers potentially eligible, examined for eligibility, confirmed eligible, included in the study, completing follow-up, and analysed	n/a
		(b) Give reasons for non-participation at each stage	n/a
		(c) Consider use of a flow diagram	n/a
Descriptive data	14*	(a) Give characteristics of study participants (eg demographic, clinical, social) and information on exposures and potential confounders	n/a
		(b) Indicate number of participants with missing data for each variable of interest	n/a
		(c) *Cohort study*—Summarise follow-up time (eg, average and total amount)	
Outcome data	15*	*Cohort study*—Report numbers of outcome events or summary measures over time	n/a
		Case-control study—Report numbers in each exposure category, or summary measures of exposure	
		Cross-sectional study—Report numbers of outcome events or summary measures	
Main results	16	(*a*) Give unadjusted estimates and, if applicable, confounder-adjusted estimates and their precision (eg, 95% confidence interval). Make clear which confounders were adjusted for and why they were included	n/a
		(*b*) Report category boundaries when continuous variables were categorized	n/a
		(*c*) If relevant, consider translating estimates of relative risk into absolute risk for a meaningful time period	n/a
Other analyses	17	Report other analyses done—eg analyses of subgroups and interactions, and sensitivity analyses	n/a
**Discussion**			
Key results	18	Summarise key results with reference to study objectives	n/a
Limitations	19	Discuss limitations of the study, taking into account sources of potential bias or imprecision. Discuss both direction and magnitude of any potential bias	n/a
Interpretation	20	Give a cautious overall interpretation of results considering objectives, limitations, multiplicity of analyses, results from similar studies, and other relevant evidence	n/a
Generalisability	21	Discuss the generalisability (external validity) of the study results	n/a
**Other information**			
Funding	22	Give the source of funding and the role of the funders for the present study and, if applicable, for the original study on which the present article is based	13

*Give information separately for cases and controls in case-control studies and, if applicable, for exposed and unexposed groups in cohort and cross-sectional studies.

Note: An Explanation and Elaboration article discusses each checklist item and gives methodological background and published examples of transparent reporting. The STROBE checklist is best used in conjunction with this article (freely available on the Web sites of PLoS Medicine at http://www.plosmedicine.org/, Annals of Internal Medicine at http://www.annals.org/, and Epidemiology at http://www.epidem.com/). Information on the STROBE Initiative is available at www.strobe-statement.org.

For peer review only

BMJ Open

Long term impact of giving antibiotics before skin incision versus after cord clamping on children born by caesarean section: protocol for a longitudinal study based on UK electronic health records

Journal:	*BMJ Open*
Manuscript ID	bmjopen-2019-033013.R1
Article Type:	Protocol
Date Submitted by the Author:	21-Aug-2019

Complete List of Authors:	Šumilo, Dana; University of Birmingham, Institute of Applied Health Research<br>Nirantharakumar, Krishnarajah; University of Birmingham, Institute of Applied Health Research & Midlands Health Data Research UK<br>Willis, Brian; University of Birmingham, Institute of Applied Health Research<br>Rudge, Gavin; University of Birmingham, Institute of Applied Health Research<br>Martin, James; University of Birmingham, Institute of Applied Health Research<br>Gokhale, Krishna; University of Birmingham, Institute of Applied Health Research<br>Thayakaran, Rasiah; University of Birmingham, Institute of Applied Health Research<br>Adderley, Nicola; Institute of Applied Health Research, University of Birmingham,<br>Chandan, Joht; University of Birmingham, Institute of Applied Health Research;<br>Okoth, Kelvin; University of Birmingham, Institute of Applied Health Research<br>Hewston, Ruth; Patient and Public contributor<br>Skrybant, Magdalena; Patient and Public contributor<br>Deeks, Jonathan; University of Birmingham, Institute of Applied Health Research; University Hospitals Birmingham NHS Foundation Trust and University of Birmingham, NIHR Birmingham Biomedical Research Centre<br>Brocklehurst, Peter; University of Birmingham, Institute of Applied Health Research

**Primary Subject Heading**:	Epidemiology
Secondary Subject Heading:	Paediatrics, Obstetrics and gynaecology
Keywords:	Caesarean section, antibiotic prophylaxis, child, Asthma < THORACIC MEDICINE, Eczema < DERMATOLOGY, immune system diseases

SCHOLARONE™
Manuscripts

Title

Long term impact of giving antibiotics before skin incision versus after cord clamping on children born by caesarean section: protocol for a longitudinal study based on UK electronic health records

Dana Šumilo[1*], Krishnarajah Nirantharakumar[1,2], Brian H Willis[1], Gavin Rudge[1], James Martin[1], Krishna Gokhale[1], Rasiah Thayakaran[1], Nicola J Adderley[1], Joht S Chandan[1], Kelvin Okoth[1], Ruth Hewston[3], Magdalena Skrybant[3], Jonathan J Deeks[1,4], Peter Brocklehurst[1]

1. Institute of Applied Health Research, University of Birmingham, Birmingham B15 2TT, UK
2. Midlands Health Data Research UK, University of Birmingham, Birmingham B15 2TT, UK
3. Patient and Public contributor
4. NIHR Birmingham Biomedical Research Centre, University Hospitals Birmingham NHS Foundation Trust and University of Birmingham, Birmingham B15 2TT, UK

*Corresponding author, e-mail: D.Sumilo@bham.ac.uk

Keywords

Caesarean section, antibiotic prophylaxis, child, asthma, eczema, immune system diseases

For peer review only

Abstract

Introduction

In the UK, about a quarter of women give birth by caesarean section and are offered prophylactic broad-spectrum antibiotics to reduce the risk of maternal postpartum infection. In 2011, national guidance was changed from recommending antibiotics after the umbilical cord was cut to giving antibiotics prior to skin incision based on evidence that earlier administration reduces maternal infectious morbidity. Although antibiotics cross the placenta, there are no known short-term harms to the baby. This study aims to address the research gap on longer term impact of these antibiotics on child health.

Methods and analysis

A controlled interrupted time series study will use anonymised mother-baby linked routine electronic health records for children born during 2006-2018 recorded in UK primary care (The Health Improvement Network, THIN, and Clinical Practice Research Datalink, CPRD) and secondary care (Hospital Episode Statistics, HES) databases. The primary outcomes of interest are asthma and eczema, two common allergy-related diseases in childhood. In-utero exposure to antibiotics immediately prior to caesarean section will be compared to no exposure when given after cord clamping. The risk of outcomes in children delivered by caesarean section will also be compared to a control cohort delivered vaginally to account for time effects. We will use all available data from THIN, CPRD and HES with estimated power of 80% and 90% to detect relative increase in risk of asthma of 16% and 18%, respectively at the 5% significance level.

Ethics and dissemination

Ethical approval has been obtained from the University of Birmingham Ethical Review Committee with scientific approvals obtained from the independent scientific advisory committees from the Medicines and Healthcare products Regulatory Agency for CPRD and the data provider, IQVIA for THIN. The results will be published in peer-reviewed journals, presented at national and international conferences, and disseminated to stakeholders.

Article Summary

Strengths and limitations of this study

- A large sample size including mother-child linked data from two nationally representative primary healthcare databases and a secondary healthcare database
- Investigation of a broad range of relevant child outcomes including severity
- Investigation of maternal outcomes using real wold evidence to confirm findings reported in randomised controlled trials
- Use of a comparison group of vaginally delivered children to effectively control for changes in diagnosis, recording and exposures over time
- Timing of prophylactic antibiotic administration is not recorded in routine healthcare data, therefore analysis is based on the estimated proportion of hospitals with the pre-incision antibiotic policy in each year during the study period

For peer review only

Introduction

Births by caesarean section (CS) account for over 20% of births globally and are increasing (1). Over one in four babies in the UK are born by CS (2-5). CS is a surgical procedure and women undergoing CS are at increased risk of developing infections after giving birth which can be prevented by prophylactic antibiotics. Before 2011, the national guidance advised administering intravenous prophylactic antibiotics for women undergoing CS after the baby's cord had been clamped to prevent exposing the baby to antibiotics. In 2011, the guidance was changed to recommend giving antibiotics to women undergoing CS prior to skin incision. This was based on evidence that earlier administration reduces maternal infectious morbidity (6). The current Cochrane review summarises data from 10 randomised trials (5,041 women) which showed a near halving of risk of all postpartum maternal infection (43%, 95% confidence interval (CI) 28-55%), endometritis (46%, CI 21-64%), and wound infection (41%, CI 19-56%) compared with giving antibiotics after clamping the baby's umbilical cord (7). Most postpartum maternal infections, however, are mild and respond well to treatment (8).

Preoperative prophylactic antibiotics rapidly cross the placenta exposing babies to high dose broad spectrum antibiotics around the time of birth (9). Although no short-term harms to the baby have been reported (6), intrapartum antibiotics have been shown to alter the gut microbiota of newborns (10). There is growing evidence that the composition of gut microbes in infants plays a role in their immune system development including response to different antigens and inflammation, and is associated with susceptibility to asthma, allergies and other immune-related diseases later in life (11-18). There is a paucity of research regarding the longer term effect of pre-incision prophylactic antibiotics for CS on child health.

Aim

The overall aim of this research study is to investigate whether the change in the guidance from recommending prophylactic antibiotics after cord clamping to pre-incision antibiotics has had any effect on the incidence of allergic and other related health conditions in children born by CS in the UK. This study will provide evidence on long term impacts of CS preoperative prophylactic antibiotics to inform current guidance regarding the timing of administration of these antibiotics. It will either reinforce the current recommendation or, if negative impacts on child health are observed, will enable assessment of the magnitude of the risks against the benefits of reduced maternal morbidity.

Objectives

The primary objective of the study is to investigate whether pre-incisional in-utero exposure to antibiotics immediately prior to birth (Intervention) compared to no pre-incisional antibiotic exposure (Comparator) increases the risk of 1) asthma and 2) eczema (Outcomes) in children born by CS (Population). The relationship between antibiotic exposure and asthma and eczema severity (defined based on prescribing information and hospital admission data) will also be explored.

For peer review only - http://bmjopen.bmj.com/site/about/guidelines.xhtml

Secondary objectives:

1. Investigating the effect of pre-incision prophylactic antibiotics in children born by CS on: a) other allergic and allergy-related diseases; b) autoimmune diseases; c) infections and inflammation; d) other immune system-related conditions; e) neurodevelopmental conditions; f) less specific measures of child health (colic and failure to thrive).

2. Investigating the effect of pre-incision prophylactic antibiotics in children born by CS on health service utilisation (overall consultation frequency in primary care and hospital admissions).

3. Investigating if the effects of a reduction in post-partum maternal infectious morbidity shown in randomised controlled trials outside the UK can be replicated in the UK using routine healthcare data.

Methods and analysis

Study design

To address the primary objective and secondary objectives 1 and 2, a controlled interrupted time series study will be undertaken using a cohort of women and their children born between 2006 and 2018 in the UK who are included in two routine primary care databases, The Health Improvement Network (THIN) or Clinical Practice Research Datalink (CPRD), and the secondary care Hospital Episode Statistics (HES) database.

Target population

Children born by CS and exposed, in utero, to antibiotics immediately prior to birth will be compared with children born by CS and not exposed, in utero, to antibiotics immediately prior to birth. Children born vaginally during the same time period will be included as a control group.

Eligibility criteria

All liveborn children for whom the birth year is between 2006 and 2018 will be included; the child and their mother's healthcare record can be linked in primary care (THIN or CPRD) or secondary care (HES) databases; the mode of delivery, CS or vaginal delivery (VD), can be identified based on recording in primary care (THIN, CPRD) and/or secondary care (HES).

Exclusion criteria

Children with missing delivery information will be excluded. In case of multiple births (e.g. twins), one of the children will be randomly selected for inclusion to ensure independence of observations.

Study outcomes

The primary outcomes for the study are the incidence of 1) asthma and 2) eczema. The main analysis for primary outcomes will be done separately in the primary care dataset and the

For peer review only - http://bmjopen.bmj.com/site/about/guidelines.xhtml

For peer review only

secondary care (HES) dataset (the latter including only hospitals for which the year of antibiotic prescribing policy change is known).

Secondary outcomes are other allergic and allergy-related diseases, autoimmune diseases, infections and inflammation, other immune system-related conditions, neurodevelopmental conditions, less specific measures of child health, healthcare utilisation, and maternal postpartum infectious morbidity (Table 1).

Table 1. The list of secondary outcomes.

Outcome	Corresponding secondary objective	Datasets analysed	
		Primary care	Secondary care
**Health conditions and symptoms in children**	1.		
Other allergic and allergy-related conditions:	1.a		
• food allergy/intolerance		x	
• allergic rhinitis and conjunctivitis		x	
• >1 allergy related disease (asthma, eczema, food allergy/intolerance, allergic rhinitis and conjunctivitis)		x	
• penicillin allergy*		x	
• anaphylaxis*		x	x
• high risk of anaphylactic reaction (prescribing of automatic injection devices containing adrenaline)*		x	
Autoimmune diseases:	1.b		
• type 1 diabetes*		x	x
• coeliac disease*		x	x
• juvenile idiopathic arthritis*		x	x
• scleroderma/systemic sclerosis*†		x	x
• inflammatory myopathies*†		x	x
• systemic lupus erythematosus (SLE)*†		x	x
• autoimmune (idiopathic) thrombocytopenic purpura (ITP)*		x	x
• juvenile pernicious (megaloblastic) anaemia*		x	x
• childhood vitiligo*†		x	
Infections and inflammation:	1.c		
• neonatal sepsis (early and late onset)			x
• other sepsis*			x
• wheeze		x	
• upper respiratory tract infections*		x	
• lower respiratory tract infections*		x	x
• bronchiolitis*		x	x
• gastroenteritis*		x	x
• inflammatory bowel disease†		x	x
• urinary tract infections*		x	x
• antibiotic prescribing*		x	
Other immune system-related conditions:	1.d		
• necrotising enterocolitis			x
• leukaemia*†		x	x

For peer review only - http://bmjopen.bmj.com/site/about/guidelines.xhtml

Neurodevelopmental conditions:	1.e		
• cerebral palsy		x	
• autism spectrum disorder*		x	
• attention deficit hyperactivity disorder (ADHD)*		x	
Less specific measures of child health:	1.f		
• colic*		x	
• failure to thrive*		x	
**Healthcare utilisation in children**	2.		
• primary care consultations*		x	
• hospital admissions*			x
**Maternal outcomes** (six weeks post-partum)	3.		
• composite infectious morbidity (wound infection, endometritis/endomyometritis, pelvic abscess, maternal sepsis, death attributed to infection)		x	x
• endometritis/endomyometritis		x	x
• wound infection		x	x
• urinary tract infection/cystitis/pyelonephritis		x	x
• sepsis		x	x
• pelvic abscess		x	x
• maternal death (if infection related)*†			x
• antibiotic prescribing*		x	
• length of hospital stay*			x

* Exploratory outcome due to insufficient evidence base, including lack of longitudinal studies investigating the association between microbiota/early antibiotic exposure and outcome of interest; †Tabulation if the outcome is very rare.

Data sources

To maximise the sample size, we will combine two UK-wide primary care research databases, THIN and CPRD, containing anonymised patient records of over 10% of the UK patient population (19). Both databases are broadly generalisable to the UK population in terms of demographics and medical condition prevalence (20, 21). There is overlap between the databases at general practice level, with THIN and CPRD containing 37% and 46% unique practices, respectively. The databases do not use the same identifiers for patients or practices, but the overlapping practices can be identified reliably using patient registration, demographic and medical record information, and the duplicates removed to create a combined THIN-CPRD dataset (19, 22).

Information on mothers and their children in the THIN-CPRD dataset can be linked using the family identification code, pregnancy codes, mother's registered or estimated delivery date, child's month of birth, and gestational age at delivery. This is the optimal linkage method allowing identification of a large proportion of mother-child pairs (23, 24). In addition, in both THIN and CPRD a large proportion of patients (about 30% and 60%, respectively) have linked hospital record data. Our estimates using THIN suggest that whilst the mode of delivery is accurately recorded in primary care (98% verified against hospital records), the recording is incomplete (the delivery mode is known for 55-64% of children). The mode of delivery is well recorded in hospital records (3), therefore where linked hospital data are

For peer review only

available, this will increase the sample of children with known mode of delivery where this is missing in primary care data.

To allow us to investigate more severe outcomes of interest requiring hospital admissions which are better recorded in secondary care, we will also create a mother-child linked database using anonymised Hospital Episode Statistics (HES) data collected for all NHS hospital admissions in England (25). This is a complex task requiring considerable expertise in record linkage, because in the UK there is no shared identifier to link maternal and child records in HES. It is, however, possible using deterministic and probabilistic linkage to attribute up to 98% of baby and mother secondary care records, as has been demonstrated in other large-scale studies of maternal and early life course research (26, 27).

We have a proposed linkage strategy which has already been validated by another recent study using matching algorithms based on HES data using organisation codes, admission dates, birth dates, GP practice codes, sex, gestation and maternal age plus a number of other variables common to birth and maternity records. The database remains nationally representative for the main birth characteristics (such as gestational age, birthweight, sex and maternal age) (27). The final output of this process will be a linked HES data set in which details of birth events and subsequent admissions of the children associated with these events can be elucidated.

HES alone, however, cannot be used to identify timing of prophylactic antibiotic administration and prophylactic antibiotics given. We will obtain the time point after which pre-incision antibiotic policy was introduced in each hospital from a national survey of maternity care providers in the UK. All maternity units undertaking caesarean sections were included in the survey with a target response rate of 85%.

The exposure and outcome measures in the healthcare databases will be defined using the Read clinical code classification system used in primary care, and International Statistical Classification of Diseases and Related Health Problems 10th Revision (ICD-10) used for clinical diagnoses, the Office of Population Censuses and Surveys Classification of Surgical Operations and Procedures 4th revision (OPCS) for procedures and Healthcare Resource Groups (HRG) codes used in HES.

Recording of some variables in healthcare data, such as breastfeeding, is incomplete; we will therefore also investigate the trends in these variables by the mode of delivery using additional data sources such as the National Maternity Surveys (28).

Methods

We will compare rates of diagnosis of asthma and other outcomes of interest over time in children born by CS, comparing outcomes according to whether each mother received pre-incisional antibiotics.

In the primary analysis we will estimate a probability that each mother received pre-incisional antibiotics according to year of birth, based on national policy uptake rates in the year of delivery for primary care data. For secondary care data, we will use the response from each hospital indicating the year of local policy implementation.

For peer review only - http://bmjopen.bmj.com/site/about/guidelines.xhtml

The major threat to validity in this observational comparison is not from case-mix confounders (indications for and incidence of CS have changed little over the study period); rather they relate to temporal changes in diagnosis and in the recording of outcomes and other exposures which impact on the number of cases identified in routine data. Patterns of diagnosis of childhood asthma, for example, have changed over time, in part driven by the revisions in the national asthma management guideline and the potentially conflicting compliance and prevalence issues faced in meeting specific indicators of the Quality and Outcomes Framework (QOF) introduced in 2004 (29, 30).

An analysis reliant on adjustment for confounding factors is unlikely to succeed in controlling for these changes as it is unclear (a) what all the drivers of all these changes have been, (b) whether any covariates exist which accurately describe these changes without substantial missing data, and (c) challenges in specification of a functional form for the relationship between these covariates and outcome.

In order to control for such temporal changes, we will use vaginally delivered (VD) children as a comparator, as this group will not have routinely received prophylactic antibiotics, but will have been subject to all the same temporal changes as those born by CS. Our study design will model the incidence of outcomes pre-intervention to predict the difference in disease incidence between babies born by CS (with antibiotics post cord-clamping) and VD (these are known to differ for some outcomes, such as asthma). From the period in which pre-incisional antibiotics are introduced, we will compare the observed incidence rate in CS children with a counterfactual incidence rate created by adding the VD-CS (post cord-clamping) difference to the observed incidence rate in VD children born post-intervention. Subject to the assumption that the model of the difference between CS and VD rates is transferable across the time periods, differences between the observed and counterfactual CS event rates will be interpreted as likely to be caused by the change in practice.

Model validity

To assess model validity, we will explore changes in the case-mix of covariates over time in relation to delivery mode. Both maternal and child characteristics will be explored. The maternal characteristics considered will be: age at childbirth, ethnicity, parity, smoking status, body mass index (BMI) before pregnancy, area deprivation coding, long term allergy-related health conditions (asthma, eczema, allergic rhinitis and conjunctivitis), pregnancy and labour complications (premature rupture of membranes, post-partum haemorrhage, manual placental removal/retained products of conception), and antibiotic prescribing during pregnancy. The characteristics of the child considered include: gestational age, sex, ethnicity, birthweight, breastfeeding status, and antibiotic prescribing during the first five years.

Estimates of sample size and statistical power

To obtain estimates of statistical power, and to estimate the impact of misclassification on estimates of increase in risk with prophylactic antibiotics before CS, we simulated the study (a) based on our estimates using the THIN database regarding the number of children with linked maternal data and asthma diagnosis rates in each year group between birth and 5

For peer review only - http://bmjopen.bmj.com/site/about/guidelines.xhtml

years of age, in line with previously published figures (31), and (b) using HES data based on estimates of children with linked maternal data and rates of children newly hospitalised for asthma assuming a readmission rate during the follow up period of 50% based on HES statistics (25).

In each simulation we created a dataset for the whole study, with 13 birth cohorts from 2006 to 2018, and follow-up included across the first 5 years of life (curtailed at the end of 2018) (Table 2). Each birth was classified by mode of delivery, and for those delivered by CS, whether antibiotics were given before skin incision, generated randomly using a binomial random number generator using an underlying probability of exposure to pre-incision antibiotics during that year of birth.

Table 2. The number of births, years of follow-up and expected events in each simulation.

	THIN-CPRD database	HES database
**CS births**	206,615	2,070,500
Post-clamping antibiotics	111,508	1,115,670
Pre-incision antibiotics	95,107	954,830
**VD births**	570,774	5,973,100
**Total births**	777,389	8,043,600
**CS person years of follow-up**	792,265	8,661,832
Post-clamping antibiotics	501,401	5,524,890
Pre-incision antibiotics	290,864	3,136,942
**VD person years of follow-up**	2,215,405	25,339,526
**Total person years of follow-up**	3,007,670	34,001,358
**New events in children born by CS**	7,173	15,333
Post-clamping antibiotics	5,324	10,454
Pre-incision antibiotics	1,849	4,880
**New events in children born by VD**	20,378	44,906
**Total events**	27,551	60,240
**Average event rate per 1000 person years**	9.2	1.8

For peer review only

Outcome events were randomly simulated according to the year-age event rates using a binomial random number generator, with increased rates in all those delivered by CS, and increased further in those who received antibiotics before skin incision. A risk ratio of 1.2 was used for increased risk of asthma with CS (32), and then further increases with risk ratios from RR=1.10 to RR=1.20 (increasing in steps of 0.02) for the increase with antibiotics before skin incision rather than after cord-clamping.

Simulations were repeated 1000 times, and statistical power estimated by noting the proportion of simulations for which the lower limit of the 95% confidence interval for the variable indicating whether antibiotics were given before skin incision was greater than a risk ratio of 1. We also recorded the estimates of the relative risk to assess attenuation bias created by misclassification.

For peer review only - http://bmjopen.bmj.com/site/about/guidelines.xhtml

The model which we fitted to analyse the simulation data included a trend term for the probability of receiving pre-incisional antibiotics with values 2006-2009=0, 2010=0.2, 2011=0.4, 2012=0.6, 2013=0.8, 2014-2018=1 with a zero value for those delivery vaginally (in the final analysis we will utilise probabilities for each year obtained from the survey).

For the primary care data we have 80% power of detecting a 16% relative increase in risk of asthma and over 90% power of detecting an 18% relative increase in risk, and being able to estimate them with a maximum of 15% underestimation from misclassification. For the HES admission data, we have over 80% power to detect a 10% relative increase in risk of asthma and 90% power to detect a 12% relative increase in risk with similar rates of underestimation due to misclassification.

The study will also be adequately powered to detect differences in the other primary outcome of interest (eczema) as incidence of GP diagnosed eczema is higher than asthma incidence in children in the UK (33-35).

Analysis

The primary and secondary outcomes will be analysed using a Poisson regression model to estimate the relative risk of developing each outcome with pre-incision compared to post-cord clamping antibiotics. We will assess for over-dispersion and if high, consider other models, such as a negative binomial. Appropriate considerations will be made to allow for the auto-correlation of data. We will look at the autocorrelation and partial autocorrelation plots to ensure any autocorrelation is accounted for. An adjustment for calendar time will be included in the model to allow for season effects. We will include terms for year, age, and the interaction between them and mode of delivery (CS or vaginal). The key outcome parameter will be estimated by an additional term to identify those who receive pre-incision rather than post-cord clamping antibiotics.

Rather than being described in dichotomous form, we will estimate the probability of pre-incision antibiotics using data from the national survey and known hospital policy. The estimated coefficient will provide an estimate of the change in policy, adjusting for misclassification.

Sensitivity analyses

Sensitivity to population changes:

- Analysis assessing the impact of the timing of the prophylactic antibiotic policy change, including comparison of analysis restricted to the years 2006-2010 (before the change in the NICE guideline) compared to years where over 50% of hospitals had introduced the policy;

- Analysis of the primary outcomes in the full HES dataset (including data for the hospitals that do not respond to the survey and therefore preclude us linking information about prophylactic antibiotic policy at hospital level) using the estimated probability of introduction of pre-incisional antibiotics according to calendar year, to investigate the consistency of findings;

For peer review only - http://bmjopen.bmj.com/site/about/guidelines.xhtml

- Analysis investigating the impact of the data recording quality (restricted to HES-linked records in THIN-CPRD database as the most accurate source of records for the mode of delivery);

- Exploratory sensitivity analysis employing the discordant sibling approach (restricting the analysis to women who gave birth by CS more than once during the study period including before and after the change in the prophylactic antibiotic policy compared to women who gave birth by VD more than once during the study period) to control further for family-related genetic and environmental factors.

Sensitivity to model changes:

- Analysis exploring whether the results are robust to the inclusion of a random effect for hospital.

Subgroup analyses:

- Exploratory subgroup analysis in HES mother-child linked database by prophylactic antibiotic type administered according to the individual hospital policies to investigate the potential impact of different antibiotics (cefuroxime alone, co-amoxiclav alone, cefuroxime + metronidazole) on child outcomes;
- Exploratory subgroup analysis by the type of CS (it is hypothesised that children delivered by elective CS have a higher likelihood of asthma and related outcomes and are more likely to be exposed to in-utero antibiotics for longer than children born to women having an emergency CS).

Patient and public involvement (PPI)

We have involved the public throughout the development of this study. This has reconfirmed the importance of the research question, particularly: the importance of assuring the baby's health as a main priority when deciding on delivery options; that uncertainty as to whether antibiotics given around the time of birth have an impact on children later in life should be resolved; that a robust study design is required to ensure the validity of the findings; a broad scope of important health and other outcomes which need to be considered; that the project needs to clearly communicate findings in terms of risks and benefits; that the findings regarding prophylactic antibiotics for CS should form part of the wider discussion regarding risks and benefits of medications in pregnancy.

Two lay parent representatives are members of our Project Management Group and an independent parent representative is a member of the Project Steering Group. We also held two PPI discussion groups with mothers and mothers-to-be in two different locations in the West Midlands. These women were from a range of backgrounds, including women from black and minority ethnic communities, a group often under-represented in research. The focus of the sessions was on exploring what women wanted to know about this research and particularly which health conditions in relation to this study were important to them. PPI helped us to confirm that we should look at a wide range of outcomes and also consider the severity of outcomes. In addition, a wider public consultation took place via a survey, a

For peer review only - http://bmjopen.bmj.com/site/about/guidelines.xhtml

For peer review only

link to which was sent to the Royal College of Obstetricians & Gynaecologists (RCOG) Women's Voices Involvement Panel and British Intrapartum Care Society (BICS) which includes lay members. Based on findings from the PPI workshops and the survey, we have added neurodevelopmental conditions as secondary/exploratory study outcomes.

Clear communication and publicising of key findings and messages are priorities of the study. Another PPI workshop is planned towards the end of the project to co-produce messages for dissemination via clinical networks, patient organisations and the media.

Ethics and dissemination

Ethical approval for this study has been provided by the Ethical Review Committee of the University of Birmingham (ERN_17-1675). The THIN database was approved by the NHS South-East Multi-Centre Research Ethics Committee (36). Approval for the use of THIN and HES-linked data in this study was provided by the Independent Scientific Ethical Advisory Committee - Scientific Review Committee panel of the data provider, IQVIA (18THIN047). The CPRD has ethics approval for observational research using anonymised data from a National Research Ethics Committee (37). The use of CPRD for this study has been approved by the Independent Scientific Advisory Committee for MHRA Database Research (18_181AR2). The use of the HES database is exempt from NHS Research Ethics Committee approval because it involves the analysis of an existing dataset of non-identifiable data. Approval for the use of HES data was obtained as part of the standard NHS Digital data approval process (38). Health Research Authority (HRA) have confirmed that as the study involves linking anonymised patient data from established databases for our study only, HRA approval is not required.

The main aim of dissemination for this project is to ensure that parents-to-be and clinicians have clear information about the benefits and risks of pre-incision prophylactic antibiotics for CS based on the latest evidence to facilitate shared decision making. We will engage with the clinical and lay stakeholders throughout the project to benefit from the wider stakeholder input, to maximise the dissemination opportunities, and to ensure that the research findings are communicated as widely as possible.

This will be achieved by: organising a further PPI workshop to produce a lay summary of the findings for wider dissemination, a dissemination event at the end of the project with lay, clinical stakeholders and professional organisations; dissemination to the clinical directors of maternity units; conference presentations, peer reviewed publications, and dissemination via website and social media.

We will also maximise dissemination through: the Collaborations for Leadership in Applied Health Research and Care (CLAHRC) West Midlands (and its successor Applied Research Collaboration ARC) making use of their platform for dissemination; our strategic alliance, Birmingham Health Partners (BHP), which aligns three NHS trusts in the West Midlands area; the West Midlands Academic Health Science Network (AHSN) whose responsibility it is to adopt, diffuse and disseminate innovation in the NHS.

For peer review only - http://bmjopen.bmj.com/site/about/guidelines.xhtml

For peer review only

Authors' contributions

DS, PB, JD, KN, BW, MS and RH participated in the conception and the initial design of the study, with further substantial contributions from GR, KG, NA, JM, RT, JS and KO. All authors participated in drafting the protocol and have approved the final version.

Acknowledgements

This study will be based in part on data from the Clinical Practice Research Datalink obtained under licence from the UK Medicines and Healthcare products Regulatory Agency and The Health Improvement Network obtained under licence from IQVIA. The data is provided by patients and collected by the NHS as part of their care and support. The interpretation and conclusions contained in this study will be those of the authors alone.

The OPCS Classification of Interventions and Procedures, codes, terms and text is Crown copyright (2016) published by Health and Social Care Information Centre, also known as NHS Digital and licenced under the Open Government Licence available at www.nationalarchives.gov.uk/doc/open-government-licence/open-government-licence.htm. Hospital Episode Statistics reused with the permission of the NHS Digital, under a data sharing agreement.

Funding

This study is funded by the National Institute for Health Research (NIHR) Health Technology Assessment (HTA) programme (16/150/01). The views expressed are those of the author(s) and not necessarily those of the NIHR or the Department of Health and Social Care.

Competing interests

None declared.

Data availability

Data for this study will be derived from THIN and CPRD primary care and linked data obtained under license from IQVIA World Publications Ltd and UK Medicines and Healthcare products Regulatory Agency. A secondary care database will be derived from HES data obtained under licence from the Health and Social Care Information Centre (NHS Digital). Data for similar cohorts can be requested from IQVIA World Publications Ltd, the UK Medicines and Healthcare Products Regulatory Agency and NHS Digital subject to protocol approval and license agreements.

References

1. Boerma T, Ronsmans C, Melesse DY, Barros AJD, Barros FC, Juan L, et al. Global epidemiology of use of and disparities in caesarean sections. Lancet. 2018;392(10155):1341-8.
2. Information Services Division Scotland. Births in Scotish Hospitals [Available from: https://www.isdscotland.org/Health-Topics/Maternity-and-Births/Births/].
3. NHS Digital. NHS Maternity Statistics, England [Available from: https://digital.nhs.uk/data-and-information/publications/statistical/nhs-maternity-statistics].

For peer review only - http://bmjopen.bmj.com/site/about/guidelines.xhtml

4. Public Health Agency of Northern Ireland. Children's Health in Northern Ireland [Available from: https://www.publichealth.hscni.net/directorates/operations/statistics].
5. Welsh Government. Maternity statistics [Available from: https://gov.wales/maternity-statistics].
6. National Collaborating Centre for Women's and Children's Health. Caesarean section. NICE Clinical guideline CG132. National Institute for Health and Care Excellence; 2011.
7. Mackeen AD, Packard RE, Ota E, Berghella V, Baxter JK. Timing of intravenous prophylactic antibiotics for preventing postpartum infectious morbidity in women undergoing cesarean delivery. The Cochrane database of systematic reviews. 2014(12):CD009516.
8. Bailey SR, Field N, Townsend CL, Rodger AJ, Brocklehurst P. Antibiotic prophylaxis for women undergoing caesarean section and infant health. BJOG : an international journal of obstetrics and gynaecology. 2016;123(6):875-6.
9. Sutton AL, Acosta EP, Larson KB, Kerstner-Wood CD, Tita AT, Biggio JR. Perinatal pharmacokinetics of azithromycin for cesarean prophylaxis. Am J Obstet Gynecol. 2015;212(6):812 e1-6.
10. Azad MB, Konya T, Persaud RR, Guttman DS, Chari RS, Field CJ, et al. Impact of maternal intrapartum antibiotics, method of birth and breastfeeding on gut microbiota during the first year of life: a prospective cohort study. BJOG : an international journal of obstetrics and gynaecology. 2016;123(6):983-93.
11. Lawley TD, Walker AW. Intestinal colonization resistance. Immunology. 2013;138(1):1-11.
12. Murgas Torrazza R, Neu J. The developing intestinal microbiome and its relationship to health and disease in the neonate. Journal of perinatology : official journal of the California Perinatal Association. 2011;31 Suppl 1:S29-34.
13. Honda K, Littman DR. The microbiome in infectious disease and inflammation. Annu Rev Immunol. 2012;30:759-95.
14. Penders J, Thijs C, van den Brandt PA, Kummeling I, Snijders B, Stelma F, et al. Gut microbiota composition and development of atopic manifestations in infancy: the KOALA Birth Cohort Study. Gut. 2007;56(5):661-7.
15. Tamburini S, Shen N, Wu HC, Clemente JC. The microbiome in early life: implications for health outcomes. Nat Med. 2016;22(7):713-22.
16. Bisgaard H, Bonnelykke K, Stokholm J. Immune-mediated diseases and microbial exposure in early life. Clin Exp Allergy. 2014;44(4):475-81.
17. Neu J, Rushing J. Cesarean versus vaginal delivery: long-term infant outcomes and the hygiene hypothesis. Clin Perinatol. 2011;38(2):321-31.
18. Jandhyala SM, Talukdar R, Subramanyam C, Vuyyuru H, Sasikala M, Nageshwar Reddy D. Role of the normal gut microbiota. World J Gastroenterol. 2015;21(29):8787-803.
19. Petersen I, McCrea R, Sammon C, Osborn D, Evans S, Cowen P, et al. Risks and benefits ofpsychotropic medication in pregnancy: cohort studies based on UK electronic primary care health records. Health Technology Assessment. 2016;20(23).
20. Blak BT, Thompson M, Dattani H, Bourke A. Generalisability of The Health Improvement Network (THIN) database: demographics, chronic disease prevalence and mortality rates. Inform Prim Care. 2011;19(4):251-5.
21. Williams T, van Staa T, Puri S, Eaton S. Recent advances in the utility and use of the General Practice Research Database as an example of a UK Primary Care Data resource. Ther Adv Drug Saf. 2012;3(2):89-99.
22. Cai B, Xu W, Bortnichak E, Watson DJ. An algorithm to identify medical practices common to both the General Practice Research Database and The Health Improvement Network database. Pharmacoepidemiol Drug Saf. 2012;21(7):770-4.
23. Cea-Soriano L, Rodriguez LAG, Cantero OF, Hernandez-Diaz S. Challenges of using primary care electronic medical records in the UK to study medications in pregnancy. Pharmacoepidem Dr S. 2013;22(9):977-85.

For peer review only - http://bmjopen.bmj.com/site/about/guidelines.xhtml

24. Charlton R, Snowball J, Sammon C, de Vries C. The Clinical Practice Research Datalink for drug safety in pregnancy research: an overview. Therapie. 2014;69(1):83-9.
25. NHS Digital. Hospital Episode Statistics. [Available from: http://content.digital.nhs.uk/hes].
26. Davidson R, Roberts SE, Wotton CJ, Goldacre MJ. Influence of maternal and perinatal factors on subsequent hospitalisation for asthma in children: evidence from the Oxford record linkage study. BMC Pulm Med. 2010;10:14.
27. Harron K, Gilbert R, Cromwell D, van der Meulen J. Linking Data for Mothers and Babies in De-Identified Electronic Health Data. PLoS One. 2016;11(10):e0164667.
28. National Perinatal Epidemiology Unit. National maternity surveys. [Available from: https://www.npeu.ox.ac.uk/maternity-surveys].
29. British Thoracic Society. British guideline on the management of asthma. Scottish Intercollegiate Guidelines Network 2016.
30. NHS Digital. Quality and Outcomes Framework [Available from: http://content.digital.nhs.uk/qof].
31. British Lung Foundation. Asthma statistics [Available from: https://statistics.blf.org.uk/asthma].
32. Thavagnanam S, Fleming J, Bromley A, Shields MD, Cardwell CR. A meta-analysis of the association between Caesarean section and childhood asthma. Clin Exp Allergy. 2008;38(4):629-33.
33. Pols DH, Wartna JB, Moed H, van Alphen EI, Bohnen AM, Bindels PJ. Atopic dermatitis, asthma and allergic rhinitis in general practice and the open population: a systematic review. Scand J Prim Health Care. 2016;34(2):143-50.
34. Punekar YS, Sheikh A. Establishing the incidence and prevalence of clinician-diagnosed allergic conditions in children and adolescents using routinely collected data from general practices. Clin Exp Allergy. 2009;39(8):1209-16.
35. Ban L, Langan SM, Abuabara K, Thomas KS, Abdul Sultan A, Sach T, et al. Incidence and sociodemographic characteristics of eczema diagnosis in children: A cohort study. J Allergy Clin Immunol. 2018;141(5):1927-9 e8.
36. NHS Health Research Authority. The Health Improvement Network (THIN) database [Available from: https://www.hra.nhs.uk/planning-and-improving-research/application-summaries/research-summaries/the-health-improvement-network-thin-database/].
37. Clinical Practice Research Datalink. Safeguarding patient data [Available from: https://www.cprd.com/safeguarding-patient-data].
38. NHS Digital. Obtaining data from NHS Digital from health research - a guide for researchers. 2016.

For peer review only

STROBE Statement—checklist of items that should be included in reports of observational studies

	Item No	Recommendation	Page No
**Title and abstract**	1	(*a*) Indicate the study's design with a commonly used term in the title or the abstract	2
		(*b*) Provide in the abstract an informative and balanced summary of what was done and what was found	2
**Introduction**			
Background/rationale	2	Explain the scientific background and rationale for the investigation being reported	3
Objectives	3	State specific objectives, including any prespecified hypotheses	3-4
**Methods**			
Study design	4	Present key elements of study design early in the paper	4;7-8
Setting	5	Describe the setting, locations, and relevant dates, including periods of recruitment, exposure, follow-up, and data collection	4;9
Participants	6	(*a*) *Cohort study*—Give the eligibility criteria, and the sources and methods of selection of participants. Describe methods of follow-up<br>*Case-control study*—Give the eligibility criteria, and the sources and methods of case ascertainment and control selection. Give the rationale for the choice of cases and controls<br>*Cross-sectional study*—Give the eligibility criteria, and the sources and methods of selection of participants	4, 6-7
		(*b*) *Cohort study*—For matched studies, give matching criteria and number of exposed and unexposed<br>*Case-control study*—For matched studies, give matching criteria and the number of controls per case	
Variables	7	Clearly define all outcomes, exposures, predictors, potential confounders, and effect modifiers. Give diagnostic criteria, if applicable	4-6; 8
Data sources/ measurement	8*	For each variable of interest, give sources of data and details of methods of assessment (measurement). Describe comparability of assessment methods if there is more than one group	5-7
Bias	9	Describe any efforts to address potential sources of bias	8
Study size	10	Explain how the study size was arrived at	6-7; 9
Quantitative variables	11	Explain how quantitative variables were handled in the analyses. If applicable, describe which groupings were chosen and why	10
Statistical methods	12	(*a*) Describe all statistical methods, including those used to control for confounding	7-8; 10
		(*b*) Describe any methods used to examine subgroups and interactions	11
		(*c*) Explain how missing data were addressed	11
		(*d*) *Cohort study*—If applicable, explain how loss to follow-up was addressed<br>*Case-control study*—If applicable, explain how matching of cases and controls was addressed<br>*Cross-sectional study*—If applicable, describe analytical methods taking account of sampling strategy	9-10
		(*e*) Describe any sensitivity analyses	11

Continued on next page

For peer review only - http://bmjopen.bmj.com/site/about/guidelines.xhtml

For peer review only

**Results**			
Participants	13*	(a) Report numbers of individuals at each stage of study—eg numbers potentially eligible, examined for eligibility, confirmed eligible, included in the study, completing follow-up, and analysed	n/a
		(b) Give reasons for non-participation at each stage	n/a
		(c) Consider use of a flow diagram	n/a
Descriptive data	14*	(a) Give characteristics of study participants (eg demographic, clinical, social) and information on exposures and potential confounders	n/a
		(b) Indicate number of participants with missing data for each variable of interest	n/a
		(c) *Cohort study*—Summarise follow-up time (eg, average and total amount)	
Outcome data	15*	*Cohort study*—Report numbers of outcome events or summary measures over time	n/a
		Case-control study—Report numbers in each exposure category, or summary measures of exposure	
		Cross-sectional study—Report numbers of outcome events or summary measures	
Main results	16	(*a*) Give unadjusted estimates and, if applicable, confounder-adjusted estimates and their precision (eg, 95% confidence interval). Make clear which confounders were adjusted for and why they were included	n/a
		(*b*) Report category boundaries when continuous variables were categorized	n/a
		(*c*) If relevant, consider translating estimates of relative risk into absolute risk for a meaningful time period	n/a
Other analyses	17	Report other analyses done—eg analyses of subgroups and interactions, and sensitivity analyses	n/a
**Discussion**			
Key results	18	Summarise key results with reference to study objectives	n/a
Limitations	19	Discuss limitations of the study, taking into account sources of potential bias or imprecision. Discuss both direction and magnitude of any potential bias	n/a
Interpretation	20	Give a cautious overall interpretation of results considering objectives, limitations, multiplicity of analyses, results from similar studies, and other relevant evidence	n/a
Generalisability	21	Discuss the generalisability (external validity) of the study results	n/a
**Other information**			
Funding	22	Give the source of funding and the role of the funders for the present study and, if applicable, for the original study on which the present article is based	13

*Give information separately for cases and controls in case-control studies and, if applicable, for exposed and unexposed groups in cohort and cross-sectional studies.

Note: An Explanation and Elaboration article discusses each checklist item and gives methodological background and published examples of transparent reporting. The STROBE checklist is best used in conjunction with this article (freely available on the Web sites of PLoS Medicine at http://www.plosmedicine.org/, Annals of Internal Medicine at http://www.annals.org/, and Epidemiology at http://www.epidem.com/). Information on the STROBE Initiative is available at www.strobe-statement.org.

For peer review only - http://bmjopen.bmj.com/site/about/guidelines.xhtml
For peer review only
