# Supplementary material for: Long-term impact of giving antibiotics before skin incision versus after cord clamping on children born by caesarean section: protocol for a longitudinal study based on UK electronic health records
Source: BMJ Open. 2019 Sep 26;9(9):e033013. doi: 10.1136/bmjopen-2019-033013 (PMC6773283; doi:10.1136/bmjopen-2019-033013)
Supplement: Reviewer comments [file bmjopen-2019-033013.reviewer_comments.pdf]

PEER REVIEW HISTORY

BMJ Open publishes all reviews undertaken for accepted manuscripts. Reviewers are asked to complete a checklist review form (**http://bmjopen.bmj.com/site/about/resources/checklist.pdf**) and are provided with free text boxes to elaborate on their assessment. These free text comments are reproduced below.

ARTICLE DETAILS

**TITLE (PROVISIONAL)**	Long term impact of giving antibiotics before skin incision versus after cord clamping on children born by caesarean section: protocol for a longitudinal study based on UK electronic health records
**AUTHORS**	Šumilo, Dana; Nirantharakumar, Krishnarajah; Willis, Brian; Rudge, Gavin; Martin, James; Gokhale, Krishna; Thayakaran, Rasiah; Adderley, Nicola; Chandan, Joht; Okoth, Kelvin; Hewston, Ruth; Skrybant, Magdalena; Deeks, Jonathan; Brocklehurst, Peter

This article was not externally reviewed at BMJ Open. Protocols that have been independently assessed prior to submission to BMJ Open are usually fast-tracked to publication on the grounds that further substantial changes will not be possible. This independent assessment will usually be external, independent review for both a substantial grant award from a non-commercial or government funder and ethics approval.
